# Evaluation of the anti-obesity effect of *Sambucus nigra* L. (elderberry) and *Vitex agnus-castus* L. (chasteberry) extracts in high-fat diet-induced obese rats

**DOI:** 10.3389/fphar.2024.1410854

**Published:** 2024-07-11

**Authors:** Şeyma Ulusoy, Ebrar İnal, Esra Küpeli Akkol, Mahmut Çiçek, Murat Kartal, Eduardo Sobarzo-Sánchez

**Affiliations:** ^1^ Department of Pharmacognosy and Natural Products Chemistry, Institute of Health Sciences, Bezmialem Vakif University, Istanbul, Türkiye; ^2^ Department of Pharmacognosy, Faculty of Pharmacy, Bezmialem Vakif University, Istanbul, Türkiye; ^3^ Department of Pharmacognosy, Faculty of Pharmacy, Gazi University, Ankara, Türkiye; ^4^ Bezmialem Center of Education, Practice, and Research in Phytotherapy, Bezmialem Vakif University, Istanbul, Türkiye; ^5^ Instituto de Investigación y Postgrado, Facultad de Medicina y Ciencias de la Salud, Universidad Central de Chile, Santiago, Chile; ^6^ Department of Organic Chemistry, Faculty of Pharmacy, University of Santiago de Compostela, Santiago, Spain

**Keywords:** obesity, elderberry, chasteberry, *Sambucus nigra* L., *Vitex agnus-castus* L.

## Abstract

The aim of this study was to investigate the effects of *S. nigra* L. and *V. agnus-castus* L. plants on obesity *in vivo*. Extracts were prepared from *S. nigra* leaves, flowers, fruits and from *V. agnus-castus* leaves, flowers, and fruits using 100% water and 70% ethanol. The total phenol and flavonoid contents of the extracts were quantified spectrophotometrically. The findings revealed that the ethanol extracts of *V. agnus-castus* and *S. nigra* flowers had the highest phenolic content, while the ethanol extracts of *S. nigra* flowers and *V. agnus-castus* leaves had the highest flavonoid content. Qualification and quantification of the phenolic contents of the extracts were carried out using liquid chromatography-high resolution mass spectrometry (LC-HRMS) analyses. The study investigated the effects of various extracts on plasma levels of leptin, insulin, triiodothyronine (T_3_), thyroxine (T_4_), triglycerides, high-density lipoprotein (HDL), low-density lipoprotein (LDL) and lipase enzyme in obesity-induced rats. The results showed that the ethanol extract of *V. agnus-castus* flowers, as well as the ethanol and water extracts of *V. agnus-castus* leaves, resulted in body weight reduction in rats with obesity. Additionally, these extracts were found to decrease serum levels of LDL, triglycerides, leptin, lipase, TNF-α, and IL-1β while increasing levels of HDL and adiponectin. The LC-HRMS results demonstrated that all three extracts exhibited relatively high concentrations of luteolin-7-glycoside and kaempferol, in comparison to the other extracts. The ethanol extract of *V. agnus-castus* flowers contained 653.04 mg/100 g of luteolin-7-glycoside and 62.63 mg/100 g of kaempferol. The ethanol extract of *V. agnus-castus* leaves contained 1,720.26 mg/100 g of luteolin-7-glycoside and 95.85 mg/100 g of kaempferol. The water extract of *V. agnus-castus* leaves contained 690.49 mg/100 g of luteolin-7-glycoside and 194.41 mg/100 g of kaempferol. The study suggests that the ethanol extract of *V. agnus-castus* flowers and leaves, as well as the water extract of *V. agnus-castus* leaves, may have potential benefits in treating obesity. However, further controlled clinical studies are necessary to evaluate the clinical efficacy of *V. agnus-castus* in treating obesity and investigate the *in vivo* anti-obesogenic effects of luteolin-7-glycoside and kaempferol separately, both in their pure form and in combination.

## 1 Introduction

Obesity is a major global public health concern of today, the prevalence of which has increased significantly in the last 50 years (Wahabi et al., 2023). The prevalence of obesity is impacted by an imbalance between energy expenditure and food consumption. Being overweight or obese increases one’s chance of developing severe chronic disorders, including but not limited to type II diabetes, hypertension, myocardial infarction, stroke, and several types of cancer (Kucukkurt et al., 2016). Several biochemical and metabolic factors are associated with obesity. Increased levels of triglyceride and LDL, as well as decreased levels of HDL and adiponectin accompany obesity (Sikaris, 2004). At the same time, obesity and insulin resistance have a well-established positive correlation (Kumar et al., 2020). Furthermore, research has shown that both the synthesis of leptin and inflammatory cytokines are elevated in obesity (Tilinca et al., 2018). In addition, it is commonly established that alterations in thyroid stimulating hormone and thyroid hormones T_3_ and T_4_ are linked to obesity (Song et al., 2019).

*Sambucus nigra* L. (Viburnaceae), commonly known as European elder or black elder, is a medicinal shrub or small tree native to Europe which has also been introduced to North America, Southeast Asia, and Australia (Atkinson and Atkinson, 2002; Sala et al., 2023; WFO, 2024a). The plant’s flowers bloom from May to June and begin to bear fruit in July. The fruits fully ripen from late August to early September (Atkinson and Atkinson, 2002).

For centuries, European elder has been utilized for medicinal purposes. The existing literature reveals that Turkish traditional medicine has utilized *S. nigra* -one of the two species of the genus *Sambucus* that naturally grows in Türkiye-to treat wounds, rheumatic discomfort, the common cold and high fever (Süntar et al., 2010). According to British Pharmacopoeia 1788, elderberry syrup which is made from ripe elderberry fruits has therapeutic use against coughs, common cold, and constipation. It is reported that a decoction of dried elderberries has been used as a laxative in Germany and a tea made from fresh elderberries has been used for the same purpose in Ukraine, Poland, Czechia and Slovakia since 1887 (EMA/HMPC, 2014). The flowers of *S. nigra* are also widely used in folk medicine. In Albania, Algeria, Italy, and Spain they have been taken internally to treat bronchial illnesses, colds, and stomachaches, as well as being used as antipyretics, diuretics, digestives, anti-rheumatics (Motti et al., 2022). Dried elderflowers, Sambuci flos, have been a part of multiple pharmacopeias for a long time, and the medicinal use of the flowers has been documented in numerous monographs. According to World Health Organization (WHO) Monographs on Selected Medicinal Plants, elder flowers are diaphoretic, expectorant, and used for symptomatic treatment of the common cold (World Health Organization, 2002). European Union’s herbal monograph on elderberry flowers recognizes that decoctions, infusions, liquid extracts, and tinctures of the flowers have long been used to relieve early symptoms of the common cold (EMA/HMPC, 2018a).

Because of the phenolic compounds in *S. nigra* fruits, the plant is commercially highly valuable (Silva et al., 2017). Flavonoids are found in every part of the plant, with rutin (quercetin-3-O-rutinoside) serving as the main flavonoid in each. Quercetin, quercetin-3-glucoside (isoquercitrin), kaempferol-3-rutinoside, kaempferol-3-glucoside (astragaline) and hyperoside are some of the other flavonoids present in flowers, fruits, and leaves of the plant. In terms of phenolic acids, the plant is rich in chlorogenic acid, neochlorogenic acid, cryptochlorogenic acid (Młynarczyk et al., 2018; Ferreira et al., 2022). Mostly due to this rich phenolic composition, the plant has been discovered to have many biological activities. Studies revealed that elderflowers, elderberries, and elder leaves all have antioxidant activity while both elderflowers and elderberries also have anti-inflammatory and antidiabetic properties (Dawidowicz et al., 2006; Ho et al., 2017). Additionally, elderberry extracts have been demonstrated *in vitro* and in human clinical trials to be beneficial in reducing the duration and intensity of flu symptoms in various influenza virus strains (Torabian et al., 2019). Thus, commercially available black elderberry products are common in the forms of syrups, drops, tablets, capsules and lozenges in the dietary supplement market all around the world as well as in Türkiye.

The potential anti-obesogenic capacities of *S. nigra* berry extracts have been studied *in vivo* and the results were promising (Farrell et al., 2015; Zielińska-Wasielica et al., 2019). However, our literature search suggests that there is only one publication of an *in vivo* study regarding the potential anti-obesity effect of *S. nigra* flower extracts, results of which have shown that polar and nonpolar extracts of the plant’s flowers reduce fat accumulation in the worm *Caenorhabditis elegans* (Bhattacharya et al., 2013). To the best of our knowledge, there are no published *in vivo* studies on how *S. nigra* leaf extracts affect obesity parameters.

*Vitex agnus-castus* L. also known as the chasteberry and monk’s pepper belongs to the family Lamiaceae (Niroumand et al., 2018; WFO, 2024b). Chasteberry is a small tree or a large shrub native to the Mediterranean area and also spread throughout Central Asia, Southern Europe and North Africa (Souto et al., 2020; WFO, 2024b). The lilac-purple flowers of the plant bloom from June to early fall and the fruits ripen in the fall (Inal et al., 2023).

*V. agnus-castus* leaves and fruits have been used for medicinal purposes for more than 2500 years in ancient Egypt, Greece, Türkiye and Rome, especially in gynecologic diseases, and were already named in the works of Hippocrates, Dioscorides and Theophrastus (Chhabra and Kulkarni, 2011; Niroumand et al., 2018). In Turkish folk medicine, chasteberry was used as a diuretic, carminative, and sedative agent. Also, the decoction of the fruits and leaves used in infertility for women, hormonal disorders and respiratory tract diseases such as asthma, bronchitis, cold and flu (Inal et al., 2023). The plant’s fruit is well-known for treating female reproductive diseases, including irregular menstruation cycles, premenstrual syndrome, menopausal disorders, and mastalgia. For women suffering from premenstrual syndrome, the European Union herbal monograph suggested a daily dosage of 20 mg of dry fruit extract, which is equal to 180 mg of the herbal material or 40 drops of the liquid extract (EMA/HMPC, 2018b).

*V. agnus-castus* consists mainly of flavonoids (casticin, penduletin, apigenin, luteolin, vitexin and orientin etc.), diterpenes (rotundifuran, viteagnusin I and vitexlactam etc.), iridoids (aucubin, agnuside and agnusoside etc.) and essential oil (Jarry et al., 2003; Chen et al., 2011; Souto et al., 2020). The flavonoid casticin is the fingerprint marker to evaluate quality control of the *V.agnus-castus* fruit extract according to European Pharmacopoeia (EMA/HMPC, 2018b). The rich variety of chemical composition of *V. agnus-castus* is responsible for different biological activities. The flower, leaf and fruit extracts of the *V.agnus-castus in vitro* anti-diabetic, antioxidant and antibacterial activities were revealed. Also, both leaf and fruit extracts have anti-inflammatory and antifungal activities that have been shown by the studies (Souto et al., 2020; Berrani et al., 2021). In addition, chasteberry fruit extracts’ anti-prostate, antiepileptic, antiangiogenic and health-promoting effects on gynecological diseases such as menopause, dysmenorrhea, premenstrual syndrome have been proven by *in vivo* and in human clinical trials (Saberi et al., 2008; Ibrahim et al., 2017; Souto et al., 2020; Mendes et al., 2022).

*V. agnus-castus* fruit and leaf extracts have been investigated *in vivo* for their possible anti-obesogenic properties, and the findings were encouraging. The fruit extracts of the *V. agnus-castus*’ anti-hyperlipidemic activity have been proven in *in vivo* studies (Abu-Raghif et al., 2015; Hamza et al., 2019). Also, the decreasing effect of *V. agnus-castus* leaf extracts on TNF-α and IL-6 levels, which are associated with obesity, has been proven (Chhabra and Kulkarni, 2014). As far as we know, there are no published *in vivo* studies on *V. agnus-castus* flower extracts thus, the flower extract’ effect on obesity parameters is uncertain.

In this study, we aimed to thoroughly examine the potential effects of fruit, leaf and flower extracts of *S. nigra* and *V. agnus-castus* plants collected from Balıkesir, Türkiye, against obesity *in vivo*. The *in vivo* effect of *S. nigra* and *V. agnus*-*castus* extracts on plasma levels of some hormones (leptin, insulin, T_3_ and T_4_), lipids (triglycerides, HDL and LDL) and lipase enzyme were evaluated by our study. To the best of our knowledge, this is the first *in vivo* study conducted on elderberry leaves and chasteberry flowers.

## 2 Materials and methods

### 2.1 Chemicals and reagents

Gallic acid (≥97.5%), quercetin (≥95%), dihydrocapsaicin (≥97%), Folin & Ciocalteu′s phenol reagent (FCR), aluminum chloride (AlCl_3_, anhydrous powder, 99.99%), sodium carbonate (Na_2_CO_3_, anhydrous powder, 99.99%) ammonium acetate (CH_3_COONH_4_, reagent grade, 98%) along with high-performance liquid chromatography (HPLC) grade solvents ethanol and methanol were all purchased from Sigma-Aldrich (Germany). Formic acid (98%–100%) for analysis was purchased from EMSURE^®^ Merck (Germany).

### 2.2 Plant materials

*Sambucus nigra* flowers and leaves were collected in May and fruits (elderberries) were collected in August from Balikesir (Burhaniye) from their natural habitat. The plant materials were identified by Prof. Dr. Murat Kartal, and voucher specimens [HERA1245, HERA1246] were deposited at the Altinbas University Herbarium, Istanbul, Türkiye. The leaf and flower samples were shade-dried, and fruit samples were packed in plastic bags and stored at −18°C until further processing.

*Vitex agnus-castus* flowers and leaves were collected in -initial flowering period- July and fruits were collected in -post-flowering period- September from Balikesir (Burhaniye) from their natural habitat. They were identified by Prof. Dr. Murat Kartal, Bezmialem Vakif University, Türkiye. A herbarium specimen of the plants was deposited in the Altinbas University Herbarium [Herbarium number: HERA1057, HERA1061]. The leaf, flower and fruit samples were shade-dried and then stored in plastic bags until further processing.

### 2.3 Extraction and quantification procedures for the Bioassay

#### 2.3.1 Preparation of extracts

Six different extracts of *S. nigra* were prepared. Dried leaves and flowers were pulverized to a fine powder in a mechanical grinder, homogenized, and sieved (1 mm mesh). Elderberries were defrosted by keeping them at room temperature for 2 h and then they were crushed in a mortar. 100 g of each sample were weighed into different Erlenmeyer flasks and the process was repeated to obtain 2 flasks of 100 g of each sample. 1 L of 50% (v/v) ethanol was added to each of the three flasks that contained respectively 100 g of dried elder leaves, 100 g of dried elderflowers, and 100 g of fresh elderberries. 1 L of distilled water was added to each of the remaining three flasks. After 12 h of maceration at 25°C, the extracts were filtered through a Whatman^®^ Grade 1 filter paper, and the filtrates were collected (Radojković et al., 2021). The extracts were then evaporated one by one to dryness under reduced pressure in a Heidolph Rotary Evaporator (Germany) at 50°C.

Six different extracts of *V. agnus-castus* were prepared. Dried leaves, flowers and fruits were pulverized to a fine powder in a mechanical grinder, homogenized, and sieved (1 mm mesh). 100 g of each sample were weighed into different Erlenmeyer flasks and the process was repeated to obtain 2 flasks of 100 g of each sample. 1 L of 70% (v/v) ethanol was added to each of the three flasks that contained respectively 100 g of dried chasteberry leaves, 100 g of dried chasteberry flowers, and 100 g of chasteberry fruits. 1 L of distilled water was added to each of the remaining three flasks. After 12 h of maceration at 25°C, the extracts were filtered through a Whatman^®^ Grade 1 filter paper, and the filtrates were collected (Sağlam et al., 2007; EMA/HMPC, 2018b). The extracts were then evaporated one by one to dryness under reduced pressure in a Heidolph Rotary Evaporator (Germany) at 50°C. Table 1 reports the codes of the extracts and extraction yields.

**TABLE 1:** Extraction yields of the extracts.

Plant	Extracted part	Extraction solvent (%)	Extract code	Extraction yield (%)
*Sambucus nigra* *(Elderberry)*	Leaf	Ethanol 50	ELE	24.33
		Water 100	ELW	32.41
	Flower	Ethanol 50	EFE	22.40
		Water 100	EFW	26.29
	Fruits (berries)	Ethanol 50	EBE	15.69
		Water 100	EBW	17.03
*Vitex agnus-castus* *(Chasteberry)*	Leaf	Ethanol 70	CLE	32.32
		Water 100	CLW	27.67
	Flower	Ethanol 70	CFE	35.00
		Water 100	CFW	30.16
	Fruits	Ethanol 70	CFrE	12.77
		Water 100	CFrW	11.81

#### 2.3.2 Analysis of total phenolic and total flavonoid contents of the extracts

Total phenolic contents of the extracts were determined by the Folin-Ciocalteu method (Singleton and Rossi, 1965). To sum up, a number of dilutions of gallic acid were prepared to create a calibration curve. Test solutions of all the extracts and gallic acid dilutions were prepared. The test solutions were prepared with 104 µL of distilled water, 8 µL of the sample, 8 µL of FCR, and 80 µL of 7% Na_2_CO_3_. The test solutions were kept in the dark for 90 min. Then, a BioTek Epoch Microplate Spectrophotometer (United States) was used to detect absorbance at the wavelength of 765 nm. The total phenolic content is the number of gallic acid equivalents (Turgut et al., 2021).

Total flavonoid contents of the extracts were calculated by aluminum chloride colorimetric method (Woisky and Salatino, 1998). Analysis of propolis: some parameters and procedures for chemical quality control. Journal of apicultural research, 37 (2), 99–105.). In brief, a number of dilutions of quercetin were prepared to create a calibration curve. Test solutions of all the extracts and quercetin dilutions were prepared. The test solutions were prepared with 134 µL of distilled water, 20 µL of the sample, 6 µL of 10% AlCl_3_, and 40 µL of CH_3_COONH_4_. The test solution was kept in the dark for 10 min. Then, a BioTek Epoch Microplate Spectrophotometer (United States) was used to detect absorbance at the wavelength of 415 nm. The total flavonoid content is the number of quercetin equivalents (Turgut et al., 2021).

#### 2.3.3 Liquid chromatography-high resolution mass spectrometer (LC-HRMS) evaluation

250 mg of each dried extract was taken and dissolved in 10 mL of methanol. These were sonicated until they turned into clear solutions. Then, the solution was filtered through (0.45 µm Millipore MillexHV filter). 30 μL of internal standard dihydrocapsaicin solution from 1,000 ppm stock solution in methanol was added to each extract solution. The sample (1 mL) was placed in the vials to be ready for LC-HRMS measurements.

Thermo ORBITRAP Q-EXACTIVE (Bremen, Germany) mass spectrometry-equipped ESI ion source and Dionex LC equipment were used for the LC-HRMS measurements. The ions in the m/z 100–900 range were scanned using the instrument’s high-resolution mode, and additional mass parameters were applied as follows: capillary temperature: 320°C, aux gas heater temperature: 320°C, gas flow rate: 45, aux gas flow rate: 10, spray voltage: 3.80 kV, and Slens RF is 50. Compound separation was carried out using a Troyasil C18 column (150 × 3 mm i.d., 3.5 μm particle size, Istanbul, Türkiye). 1% formic acid in water and 1% formic acid in methanol made up the mobile phases A and B, respectively. The gradient program was 0–1.00 min 50% A and 50% B, 1.01–6.00 min 100% B, and finally 6.01–15 min 50% A and 50% B. The flow rate of the mobile phase was 0.35 mL/min, and the column temperature was set to 22°C. Environmental conditions were set as temperature 22.0°C ± 5.0°C and relative humidity (50 ± 15) % rh. Compounds were identified by comparing the retention times and target ions of the compounds in Bezmialem Vakif University’s Drug Application and Research Center (ILMER) Library (Kızıltaş et al., 2021).

### 2.4 Pharmacological experiments

#### 2.4.1 *In Vivo* biological activity tests

##### 2.4.1.1 Animals

Male Wistar albino rats weighing 200–250 g were sourced from the Saki Yenilli Experimental Animals Laboratory. The rats were kept under standard conditions with 12 h of light and 12 h of darkness and fed with standard pellet feed and water *ad libitum*. Experimental studies were conducted during the day from 10:00 to 18:00. A minimum of 7 rats were used for each group, and all studies were conducted in accordance with international ethical rules, without violating animal rights.

##### 2.4.1.2 Preparation of test samples

In biological activity experiments, the test samples were suspended in 0.5% sodium carboxymethyl cellulose (CMC) solution with ultrasonic baths, if necessary, and administered to the experimental animals at a dose of 100 mg/kg using a special gastric gauge. The control group animals were given a 0.5% CMC solution used only in the preparation of test samples. In experiments, test samples were applied to rats for 7 weeks.

##### 2.4.1.3 Reference drug

Orlistat used as the reference substance was prepared by suspension in 0.5% CMC at a dose of 5 mg/kg and was administered to animals once daily by gastric gauge during the experimental period.

##### 2.4.1.4 *In Vivo* anti-obesity activity

The experiments were continued for 7 weeks and weight tracking of the rats used was carried out at weekly intervals. The rats are housed in individually and divided into 15 groups as indicated in Table 2. The first group consisted of healthy rats fed with standard pellet feed, while the rats in other groups were fed with high fat dietary feed containing 40% beef. After a 3-days adaptation period, control group was administered 0.5% CMC suspension by gastric gavage once a day during the experiment in an amount equal to the volume of the substances to be administered in the other groups. The other high-fat diet groups were given the extract and orlistat solutions dissolved in high fat dietary feed containing 40% beef at the doses specified in once a day during the 49-day experimental period by gastric gavage administration in equal volumes with the other groups. On the 49th day, the experiment was terminated.

**TABLE 2:** Study design.

Groups	Administration
***Control group***	0.5% CMC suspension (1 mL/kg/p.o.) + Standard pellet feed
***Negative control group***	0.5% CMC suspension (1 mL/kg/p.o.) + high fat dietary containing 40% beef
***Test material groups*** ELE ELW EFE EFW EBE EBW CLE CLW CFE CFW CFrE CFrW	Test samples suspended in 0.5% CMC suspension (100 mg/kg, p.o) + high fat dietary feed containing 40% beef
***Reference drug group***	Orlistat (5 mg/kg dose) + high fat dietary containing 40% beef

ELE, 50% ethanolic extract of elderberry leaves; ELW, elderberry leaf water extract; EFE, 50% ethanolic extract of elderflowers; EFW, elderflower water extract; EBE, 50% ethanolic extract of elderberries; EBW, elderberry water extract; CLE, 70% ethanolic extract of chasteberry leaves; CLW, chasteberry leaf water extract; CFE, 70% ethanolic extract of chasteberry flower; CFW, chasteberry flower water extract; CFrE, 70% ethanolic extract of chasteberry fruit; CFrW, chasteberry fruit water extract.

#### 2.4.2 *In Vitro* activity tests

##### 2.4.2.1 Biochemical analysis

Serum parameters were measured by collecting blood samples from the heart of rats after they were killed at the end of the 7-week experimental period. The blood samples were collected in tubes without anticoagulants. After waiting for clotting at room temperature, the serum was obtained by centrifuging at 1,000 g for 10 min. The obtained serum was stored at −20 °C in 1.5 mL Eppendorf tubes until measurements were taken. Insulin (Cat. EZRMI-13K), triglycerides, HDL, LDL, adiponectin (Human Diagnostica. GmbH, Germany), leptin (Cat. EZRL-83K), free T_3_ (DSL-10-41100), free T_4_ (DSL-10-40100i), TNF-α, IL-1β, and lipase levels were measured using commercial kits in the obtained serum samples (Turgut et al., 2021).

##### 2.4.2.2 HDL cholesterol (HDL-C) measurement

The method is based on the conversion of the colorless product resulting from the reaction of non-esterified cholesterol with N, N-bis(4-sulfobutyl)-m-toluidine disodium salt (DSBmT) to a colored compound by cholesterol esterase enzyme.

Serum HDL-C levels were calculated in mg/dL using the Dimension RL Max autoanalyzer.

##### 2.4.2.3 LDL cholesterol (LDL-C) measurement

The method is based on the conversion of very low-density lipoprotein, HDL, and chylomicrons into LDL-C under specific conditions, followed by the formation of colored compounds with the help of enzymes and surfactants, and the measurement of their absorbance (Bacak, 2010).

Serum LDL-C levels were measured in mg/dL using the Hitachi Moduler autoanalyzer.

##### 2.4.2.4 Determination of triglyceride levels

The method was performed by lipoprotein lipase enzyme, which releases glycerol from triglycerides, followed by phosphorylation of glycerol with glycerol kinase to form glycerol-3-phosphate. Glycerol-3-phosphate is then oxidized by glycerol-3-phosphate oxidase enzyme to form dihydroxyacetone phosphate and hydrogen peroxide, which reacts with 4-chlorophenol in the presence of H_2_O_2_. The method is based on measuring the absorbance of the resulting color at 510 and 700 nm (Bacak, 2010).

Serum triglyceride (TG) levels were calculated in mg/dL using the Dimension RL Max autoanalyzer and the bichromate end-point technique.

##### 2.4.2.5 Determination of leptin levels

Leptin levels were measured using an ELISA kit. Reagents, samples, and standards were prepared according to the manufacturer’s instructions and added to wells, followed by incubation for 1 h. Then, 100 µL of biotinylated antibody solution was added to each well and incubated for 1 h. After washing the wells three times with the washing solution, 100 µL of streptavidin-HRP conjugate was added and incubated for 30 min, followed by washing. Substrate solution (100 µL) was added and incubated for 10 min, and finally, the reaction was stopped by adding 100 µL of stop solution.

##### 2.4.2.6 Determination of IL-1β and TNF-α levels

IL-1β and TNF-α levels were measured using ELISA kits. The reagents, samples, and standards were prepared according to the manufacturer’s instructions. 100 μL of standard and test samples were added to each well of a 96-well plate and incubated at 37°C for 2 h. The liquids were then removed from each well and 100 µL of biotin antibody was added to the wells and incubated at 37°C for 1 h. After the incubation period, the wells were washed three times with washing solution. Then, the wells were emptied and 100 µL of horseradish peroxidase (HRP)-avidin was added to each well and incubated at 37°C for 1 h. After emptying the wells, they were washed five times with washing solution. Next, 90 µL of 3,3′,5,5′-tetramethylbenzidine (TMB) substrate was added to each well and incubated in the dark at 37°C for 30 min. Finally, 50 µL of stopping solution was added and the ELISA microplate reader was used to read at 450 nm.

##### 2.4.2.7 Determination of lipase enzyme inhibition

Test samples were diluted with 0.1 M Tris-HCl buffer (pH = 8.0) to final concentrations of 25, 50, 100, 200, and 400 μg/mL in microplates. Lipase inhibition levels were carried out by a modified method using p-nitro phenyl butyrate (p-NPB) (CAS: 2635-84-9) as substrate (Zhang et al., 2008; Bustanji et al., 2011; Jo et al., 2017). Orlistat, which has lipase inhibitory effect, was used as a reference substance. Orlistat was diluted with buffer solution to final concentrations of 6.25, 12.5, 25, 50, and 100 μg/mL in microplate. Absorbance measurements of the samples were performed in a spectrophotometer using a 96-well microplate at 405 nm wavelength. Each concentration of each sample was studied in 3 parallel runs.

The percent of enzyme inhibition values determined as a result of the experiment and the logarithm (ordinate and abscissa) of the concentration to which it belongs were graphed and the IC_50_ values of the samples on lipase enzyme were determined from the graphical equation. Samples with IC_50_ values outside the studied dose range were not considered as lipase inhibitory.

#### 2.4.3 Statistical analysis

At the end of the study, weekly weights of the rats, weight difference between weeks, percent of weight changes between weeks and biochemical parameters were calculated as statistical mean ± standard deviation. GraphPad Prism 6.0 (San Diego, CA, United States) software was used for statistical analyses. ANOVA test was performed for all parameters and then Dunnett’s test was applied.

## 3 Results

### 3.1 Total phenolic and total flavonoid contents of the elderberry and the chasteberry extracts

Total phenol and flavonoid content of the extracts was calculated according to the equations as gallic acid and quercetin equivalent (mg g^−1^ extract), respectively. The results shown in Table 3 indicate that chasteberry flower 70% ethanolic extract (CFE; 68.42 ± 1.20 mg/g extract) and elderflower 50% ethanolic extract (EFE; 59.05 ± 0.21 mg/g extract) had the highest total phenol content. The extracts which had the highest flavonoid content were EFE (35.43 ± 0.23 mg/g extract) and chasteberry leaves 70% ethanolic extract (CLE; 31.99 ± 1.17 mg/g extract).

**TABLE 3:** Total phenol and flavonoid contents of the *Sambucus nigra* and *Vitex agnus-castus* extracts.

Extracts	Total phenol^a^ *contents ± S.E.M.* ^b^	Total flavonoid^c^ *contents ± S.E.M.*
**ELE**	49.20 ± 0.35	23.92 ± 0.11
**ELW**	44.86 ± 0.34	16.89 ± 0.28
**EFE**	59.05 ± 0.21	35.43 ± 0.23
**EFW**	49.38 ± 0.21	23.33 ± 0.33
**EBE**	35.49 ± 0.28	5.63 ± 0.23
**EBW**	36.84 ± 0.29	6.06 ± 0.25
**CLE**	49.11 ± 0.82	31.99 ± 1.17
**CLW**	50.78 ± 1.81	21.67 ± 1.46
**CFE**	68.42 ± 1.20	21.76 ± 1.51
**CFW**	40.89 ± 0.87	9.00 ± 0.49
**CFrE**	27.05 ± 0.79	11.21 ± 0.74
**CFrW**	22.66 ± 0.83	3.17 ± 0.17

^a^ Data expressed in mg equivalent of gallic acid to 1 g of extract.

^b^ Standard error mean (n = 3).

^c^ Data expressed in mg equivalent of quercetin to 1 g of extract.

### 3.2 LC-HRMS analysis

The qualitative and quantitative determination of phenolic compounds of *S. nigra* and *V. agnus-castus* extracts were performed with LC-HRMS, and are demonstrated in Table 4. LC-HRMS chromatograms are presented in Supplementary Figures S1–S12.

**TABLE 4:** Comparison of phenolic compounds by extracts (mg/100 g extract).

Phenolic compounds	U%	m/z	ELE	ELW	EFE	EFW	EBE	EBW	CLE	CLW	CFE	CFW	CFrE	CFrW
**Apigenin**	2.87	269.05	0.05	0.02	0.05	0.03	0.02	0.02	3.87	4.33	68.40	77.22	7.76	3.32
**Apigenin-7-glucoside**	3.59	431.10	0.07	0.05	0.50	0.18	0.06	0.02	241.42	8.81	684.02	52.04	93.53	n.d
**Caffeic acid**	3.74	179.04	19.26	50.09	80.06	n.d	0.19	n.d	40.82	145.13	63.63	107.11	61.13	0.90
**Casticin**	6.55	375.11	n.d	n.d	n.d	n.d	n.d	n.d	409.64	160.46	590.36	108.51	3,264.28	504.43
**Chlorogenic acid**	3.58	353.09	3,736.26	897.58	3,145.95	1,267.69	33.42	7.90	3,889.66	6,958.63	8,227.81	4,789.74	826.60	138.45
**Cirsilineol**	3.90	343.08	n.d	n.d	n.d	n.d	n.d	n.d	40.72	10.95	153.71	22.45	501.70	63.43
**Fumaric acid**	2.88	115.01	n.d	n.d	n.d	n.d	n.d	n.d	2,844.38	4,806.52	n.d	7,373.07	4,159.18	n.d
**Hyperoside**	3.46	463.09	188.10	24.86	575.76	272.67	66.40	46.16	9.28	3.61	22.27	1.29	13.07	0.34
**Kaempferol**	3.56	285.04	n.d	n.d	3.79	13.09	n.d	n.d	95.85	194.41	62.63	85.53	34.28	37.96
**Luteolin-7-glucoside**	4.14	447.09	0.63	0.04	n.d	n.d	0.12	0.05	1,720.26	690.49	653.04	n.d	683.61	187.04
**Naringenin**	4.20	271.06	0.08	n.d	84.29	7.14	0.26	0.24	18.12	19.61	24.46	17.35	23.97	10.90
**Penduletin**	3.20	343.08	n.d	n.d	n.d	n.d	n.d	n.d	53.30	17.68	190.05	30.90	596.98	8,453.61
**Quercetin**	2.95	301.04	2.20	5.93	24.37	136.58	1.78	41.56	4.02	2.14	2.35	1.98	14.77	2.55
**Quercitrin**	3.78	447.09	11.38	1.31	67.00	32.56	0.61	0.40	n.d	n.d	n.d	n.d	n.d	n.d
**Rutin**	3.07	609.15	3,032.09	506.56	6,235.87	1,906.35	578.30	251.37	n.d	n.d	n.d	n.d	n.d	n.d

n.d.: not detected.

As a result of LC-HRMS analysis of 50% ethanolic extract of elderberry leaves (ELE), the main phenolic components identified were chlorogenic acid (3736.26 mg/100 g), rutin (3032.09 mg/100 g) and hyperoside (188.10 mg/100 g). Chlorogenic acid (897.58 mg/100 g), rutin (506.56 mg/100 g) and caffeic acid (50.09 mg/100 g) were analyzed as the major phenolic compounds of the elderberry leaf water extract (ELW). Rutin (6,235.87 mg/100 g), chlorogenic acid (3,145.95 mg/100 g), and hyperoside (575.76 mg/100 g) were determined to be the main phenolic compounds of 50% ethanolic extract of elderflowers (EFE). Similarly, elderflower water extract’s (EFW) major phenolic compounds were identified as rutin (1,906.35 mg/100 g), chlorogenic acid (1,267.69 mg/100 g), and hyperoside (272.67 mg/100 g). Rutin (578.30 mg/100 g), hyperoside (66.40 mg/100 g) and chlorogenic acid (33.42 mg/100 g) were determined to be the main phenolic compounds of 50% ethanolic extract of elderberries (EBE). Major phenolic compounds of elderberry water extract (EBW) were determined as rutin (251.37 mg/100 g), hyperoside (46.16 mg/100 g) and quercetin (41.56 mg/100 g). The analysis results show that the major phenolic compounds determined through the ILMER LC-HRMS library in all prepared 50% ethanolic extracts of *S. nigra* (ELE, EFE, EBE) are the same 3 compounds: rutin, chlorogenic acid and hyperoside.

The 70% ethanolic extract of chasteberry leaves (CLE)’s major phenolic components were analyzed as chlorogenic acid (3,889.66 mg/100 g), fumaric acid (2,844.38 mg/100 g) and luteolin-7-glucoside (1,720.26 mg/100 g). Similarly, chasteberry leaf water extract (CLW)’s major phenolic components were detected as chlorogenic acid (6,958.63 mg/100 g), fumaric acid (4,806.52 mg/100 g) and luteolin-7-glucoside (690.49 mg/100 g) respectively. Chlorogenic acid (8,227.81 mg/100 g), apigenin-7-glucoside (684.02 mg/100 g) and luteolin-7-glucoside (653.04 mg/100 g) were analyzed as the major compounds of the chasteberry flower 70% ethanolic extract (CFE) while the fumaric acid (7,373.07 mg/100 g), chlorogenic acid (4,789.74 mg/100 g) and casticin (108.51 mg/100 g) were the main phenolic components of the chasteberry flower water extract (CFW). In the chasteberry fruit 70% ethanolic extract (CFrE)’s major phenolic compounds were detected as fumaric acid (4,159.18 mg/100 g), casticin (3264.28 mg/100 g) and chlorogenic acid (826.60 mg/100 g). Also, the chasteberry fruit water extract (CFrW)’s major phenolic compounds were analyzed as penduletin (8,453.61 mg/100 g), casticin (504.43 mg/100 g) and luteolin-7-glucoside (187.04 mg/100 g).

### 3.3 Bioactivity study results

At the end of the 7-week experiment period, the highest anti-obesity effect was observed in the orlistat group, which was the reference substance group. At the same time, when compared with the negative control group, the anti-obesity effect of the substances coded CLE, CLW and CFE was observed and it was determined that the highest anti-obesity effect was in the group in which the test sample CLE was applied (Table 5). According to the weight average results, it was determined that the average weight of the control group was 299.31 g, the negative control group was 391.28 g and the reference group was 291.12 g at the end of the 7-week experiment period. The average weights of the test samples of groups CLE, CLW and CFE were 297.43, 301.03, and 305.14 g, respectively. When the experiment was completed, it was determined that the average of the total weight of the rats according to the weight difference results was 81.64 g in the test sample group CLE, 75.17 g in the test sample group CLW and 92.67 g in the test sample group CFE. Similarly, when the general appearance of intra-abdominal adipose tissue was evaluated after the experimental period, it was observed that the obesity group contained a higher proportion of intra-abdominal fat compared to the other groups. According to the findings obtained in terms of weight, it was determined that there was a decrease in body weight in the groups belonging to test samples CLE, CLW and CFE when compared with the control group and negative control group.

**TABLE 5:** Weight averages of experimental groups according to weeks.

Groups	Mean weight (g) ± SE.M.							
	Week 0	Week 1	Week 2	Week 3	Week 4	Week 5	Week 6	Week 7
**Control**	221.40 ± 10.85	239.95 ± 12.94	249.44 ± 13.26	258.12 ± 14.61	265.34 ± 13.61	278.36 ± 12.54	289.46 ± 10.32	299.31 ± 12.44
**Negative control**	234.66 ± 12.08	251.13 ± 14.05	298.51 ± 12.19	319.26 ± 13.04	327.43 ± 11.36	366.55 ± 11.13	378.23 ± 11.01	391.28 ± 10.12
**ELE**	249.31 ± 10.01	259.81 ± 11.39	265.77 ± 11.23	279.95 ± 13.34	298.36 ± 11.35	310.52 ± 9.71	322.71 ± 8.10	353.51 ± 9.41
**ELW**	231.75 ± 10.92	243.39 ± 11.34	259.94 ± 14.21	278.45 ± 11.49	292.12 ± 12.29	305.12 ± 14.08	318.51 ± 13.12	347.05 ± 11.78
**EFE**	244.06 ± 7.34	261.78 ± 10.61	298.38 ± 9.08	302.45 ± 9.08	317.42 ± 11.46	325.61 ± 11.39	334.17 ± 9.08	358.83 ± 9.85
**EFW**	230.95 ± 11.62	241.34 ± 10.06	257.12 ± 9.94	290.62 ± 8.75	311.65 ± 9.26	326.91 ± 9.64	341.57 ± 11.12	372.11 ± 10.03
**EBE**	235.52 ± 10.14	261.30 ± 8.67	279.60 ± 9.56	296.30 ± 12.82	313.76 ± 11.08	337.74 ± 13.12	354.37 ± 12.68	381.17 ± 9.45
**EBW**	241.11 ± 11.43	259.28 ± 9.25	270.73 ± 10.42	285.53 ± 15.08	301.57 ± 14.12	337.60 ± 12.71	371.08 ± 11.26	390.46 ± 12.67
**CLE**	215.79 ± 8.64	223.64 ± 9.22	237.06 ± 10.27	246.32 ± 12.91	259.01 ± 13.34	274.34 ± 12.80	289.34 ± 11.18	297.43 ± 9.37
**CLW**	225.86 ± 9.15	230.12 ± 12.31	241.36 ± 11.07	262.11 ± 12.53	274.82 ± 12.64	289.26 ± 10.45	292.05 ± 9.76	301.03 ± 11.35
**CFE**	212.47 ± 7.48	225.58 ± 9.09	239.14 ± 9.05	248.11 ± 11.09	261.95 ± 10.12	286.03 ± 9.34	294.32 ± 9.01	305.14 ± 10.22
**CFW**	224.55 ± 9.94	242.52 ± 7.82	261.43 ± 9.52	278.47 ± 10.45	286.69 ± 11.27	291.16 ± 11.46	314.07 ± 12.33	329.27 ± 10.54
**CFrE**	233.47 ± 8.89	246.83 ± 14.13	261.24 ± 12.61	274.76 ± 13.91	288.11 ± 14.92	297.44 ± 11.92	312.64 ± 9.98	343.12 ± 9.26
**CFrW**	236.17 ± 7.23	259.78 ± 10.14	274.52 ± 9.53	285.24 ± 10.67	297.27 ± 12.91	306.72 ± 14.21	313.52 ± 12.26	355.04 ± 10.13
**Reference**	221.27 ± 6.79	229.50 ± 7.06	237.61 ± 8.01	251.13 ± 8.26	262.13 ± 8.10	284.32 ± 8.02	290.31 ± 9.12	291.12 ± 8.61

S.E.M.: standard error meaning.

ELE, 50% ethanolic extract of elderberry leaves; ELW, elderberry leaf water extract; EFE, 50% ethanolic extract of elderflowers; EFW, elderflower water extract; EBE, 50% ethanolic extract of elderberries; EBW, elderberry water extract; CLE, 70% ethanolic extract of chasteberry leaves; CLW, chasteberry leaf water extract; CFE, 70% ethanolic extract of chasteberry flower; CFW, chasteberry flower water extract; CFrE, 70% ethanolic extract of chasteberry fruit; CFrW, chasteberry fruit water extract.

In order to evaluate the dyslipidaemia associated with obesity, HDL-C, LDL-C, triglyceride, adiponectin and leptin levels secreted from adipose tissue and associated with obesity were evaluated in the sera obtained from the experimental groups. Compared to the negative control group, serum LDL-C, triglyceride and leptin levels decreased, while HDL-C and adiponectin levels increased in the test samples CLE, CLW and CFE and the reference drug group (Table 6). In our study, no statistically significant change was obtained in T_3_ and T_4_ levels in the study groups (Table 6).

**TABLE 6:** Effects of study materials on HDL-C, LDL-C, triglyceride, adiponectin, leptin, T3 and T4 levels.

Groups	HDL (mg/dL)	LDL (mg/dL)	Triglyceride (mg/dL)	Adiponectin (ng/mL)	Leptin (ng/dL)	T_3_ (pg/mL) (%)	T_4_ (ng/mL) (%)
**Control**	97.34 ± 6.72	37.09 ± 3.81	98.43 ± 5.34	12.03 ± 2.55	1.71 ± 0.83	1.42 ± 0.55	0.54 ± 0.29
**Negative control**	62.52 ± 4.17	45.40 ± 3.24	116.27 ± 6.51	9.34 ± 1.42	4.98 ± 1.56	1.30 ± 0.41	0.45 ± 0.34
**ELE**	61.16 ± 6.05	35.39 ± 5.10	91.45 ± 7.38	8.82 ± 0.67	3.24 ± 1.17	1.53 ± 0.72	0.63 ± 0.09
**ELW**	70.63 ± 5.12	39.48 ± 3.27	114.75 ± 8.91	7.49 ± 2.13	3.54 ± 0.82	1.24 ± 0.37	0.57 ± 0.11
**EFE**	65.51 ± 3.08	41.25 ± 3.86	123.51 ± 5.42	7.78 ± 0.81	3.70 ± 0.51	1.26 ± 0.65	0.59 ± 0.08
**EFW**	53.38 ± 6.49	42.05 ± 4.12	131.12 ± 5.08	8.91 ± 1.73	4.08 ± 0.94	1.42 ± 0.53	0.41 ± 0.07
**EBE**	75.92 ± 3.41	43.72 ± 4.06	112.53 ± 5.03	5.65 ± 0.79	4.67 ± 0.89	1.34 ± 0.46	0.55 ± 0.06
**EBW**	71.73 ± 7.62	45.13 ± 3.90	135.09 ± 7.42	9.25 ± 0.56	4.98 ± 0.43	1.56 ± 0.74	0.42 ± 0.02
**CLE**	94.45 ± 5.57*	31.27 ± 4.26*	72.14 ± 5.08**	12.16 ± 1.05*	1.51 ± 0.38***	1.44 ± 0.91	0.54 ± 0.05
**CLW**	93.54 ± 5.65*	32.71 ± 2.65*	83.96 ± 4.37*	12.41 ± 2.05*	1.59 ± 0.71***	1.31 ± 0.35	0.46 ± 0.03
**CFE**	82.65 ± 3.27**	31.90 ± 3.61*	83.23 ± 4.14*	10.67 ± 1.42	1.63 ± 0.51**	1.27 ± 0.47	0.33 ± 0.02
**CFW**	78.22 ± 6.51	32.46 ± 3.48*	85.01 ± 4.53*	10.63 ± 0.98	2.51 ± 0.98**	1.53 ± 0.62	0.52 ± 0.05
**CFrE**	69.14 ± 5.93	33.15 ± 4.12	94.32 ± 6.96	11.32 ± 1.74	2.72 ± 0.56**	1.34 ± 0.31	0.47 ± 0.06
**CFrW**	71.61 ± 6.05	35.28 ± 3.29	96.77 ± 5.13	9.51 ± 1.44	2.19 ± 1.05**	1.35 ± 0.54	0.31 ± 0.12
**Reference**	95.40 ± 4.19**	29.53 ± 3.41**	71.65 ± 4.82**	13.05 ± 1.63**	1.43 ± 0.82***	1.31 ± 0.29	0.39 ± 0.08

S.E.M.: standard error meaning; *: *p* < 0.05; **p < 0.01; ***: *p* < 0.001.

ELE, 50% ethanolic extract of elderberry leaves; ELW, elderberry leaf water extract; EFE, 50% ethanolic extract of elderflowers; EFW, elderflower water extract; EBE, 50% ethanolic extract of elderberries; EBW, elderberry water extract; CLE, 70% ethanolic extract of chasteberry leaves; CLW, chasteberry leaf water extract; CFE, 70% ethanolic extract of chasteberry flower; CFW, chasteberry flower water extract; CFrE, 70% ethanolic extract of chasteberry fruit; CFrW, chasteberry fruit water extract. Bold values are statistically significant.

Serum TNF-α and IL-1β levels of rats were also analyzed in order to evaluate chronic inflammation associated with obesity. Serum TNF-α and IL-1β levels were found to be lower in test samples CLE, CLW and CFE compared to the negative control group (Table 7).

**TABLE 7:** Cytokine levels of the study materials in serum in obesity model in rats.

Groups	TNF-α level (pg/mL)	IL-1β level (pg/mL)
**Control**	703.12 ± 15.84	914.82 ± 10.67
**Negative control**	925.31 ± 17.06	1,271.94 ± 11.83
**ELE**	964.27 ± 11.13	1,017.51 ± 14.06
**ELW**	917.46 ± 13.24	965.24 ± 13.21
**EFE**	944.07 ± 11.45	913.05 ± 11.72
**EFW**	890.14 ± 10.19	996.22 ± 12.09
**EBE**	851.82 ± 12.65	871.24 ± 11.75
**EBW**	804.51 ± 13.73	886.31 ± 15.49
**CLE**	613.33 ± 10.08**	722.08 ± 13.48**
**CLW**	731.19 ± 8.56	746.12 ± 11.37**
**CFE**	692.31 ± 11.25*	790.68 ± 17.08*
**CFW**	705.74 ± 10.61	810.25 ± 11.92*
**CFrE**	794.02 ± 11.26	813.05 ± 9.97*
**CFrW**	786.13 ± 9.73	820.94 ± 8.89*
**Reference**	675.09 ± 9.82*	731.28 ± 10.04**

S.E.M.: standard error meaning; *: *p* < 0.05; **p < 0.01; ***: *p* < 0.001 ***.

ELE, 50% ethanolic extract of elderberry leaves; ELW, elderberry leaf water extract; EFE, 50% ethanolic extract of elderflowers; EFW, elderflower water extract; EBE, 50% ethanolic extract of elderberries; EBW, elderberry water extract; CLE, 70% ethanolic extract of chasteberry leaves; CLW, chasteberry leaf water extract; CFE, 70% ethanolic extract of chasteberry flower; CFW, chasteberry flower water extract; CFrE, 70% ethanolic extract of chasteberry fruit; CFrW, chasteberry fruit water extract. Bold values are statistically significant.

Blood glucose levels and serum insulin levels of rats were evaluated to determine obesity-related insulin resistance. While no significant difference was observed in blood glucose levels determined by glucose meter, serum insulin levels were found to be lower in the reference substance group and in the groups administered test samples CLE, CLW and CFE compared to the negative control group (Table 8).

**TABLE 8:** Effects of study materials on blood glucose and serum insulin levels.

Groups	Blood glucose level (mg/dL)	Serum insulin level (mIU/L)
**Control**	122.70 ± 4.18	8.16 ± 1.90
**Negative control**	134.03 ± 4.96	11.93 ± 2.24
**ELE**	129.15 ± 4.04	7.08 ± 1.71
**ELW**	123.46 ± 5.07	8.08 ± 0.45
**EFE**	138.53 ± 3.74	9.46 ± 0.59
**EFW**	141.05 ± 4.09	8.92 ± 0.71
**EBE**	137.16 ± 5.12	9.30 ± 1.44
**EBW**	129.43 ± 5.72	9.61 ± 0.34
**CLE**	135.08 ± 4.62	6.89 ± 0.83*
**CLW**	149.17 ± 6.24	7.79 ± 0.37*
**CFE**	145.26 ± 9.59	6.41 ± 0.81*
**CFW**	133.07 ± 11.36	7.46 ± 1.22
**CFrE**	121.13 ± 8.68	9.49 ± 1.49
**CFrW**	129.54 ± 7.16	10.37 ± 1.18
**Reference**	125.6 ± 8.25	5.19 ± 0.34**

S.E.M.: standard error meaning; *:*p* < 0.05; **p < 0.01; ***: *p* < 0.001.

ELE, 50% ethanolic extract of elderberry leaves; ELW, elderberry leaf water extract; EFE, 50% ethanolic extract of elderflowers; EFW, elderflower water extract; EBE, 50% ethanolic extract of elderberries; EBW, elderberry water extract; CLE, 70% ethanolic extract of chasteberry leaves; CLW, chasteberry leaf water extract; CFE, 70% ethanolic extract of chasteberry flower; CFW, chasteberry flower water extract; CFrE, 70% ethanolic extract of chasteberry fruit; CFrW, chasteberry fruit water extract. Bold values are statistically significant.

When the serum lipase levels of the rats were compared, it was determined that although it was much more pronounced in the orlistat-treated group, it was lower in test samples CLE, CLW and CFE compared to the negative control group (Table 9). *In vitro* lipase enzyme inhibitory effect was observed in test samples CLE, CLW and CFE and in the orlistat group used as reference substance, while no statistically significant effect was observed in other groups (Table 9).

**TABLE 9:** Effect of study materials on IC50 levels determined on serum lipase and lipase enzyme in rats.

Groups	Serum lipase level (U/L)	Lipase enzyme inhibition (IC_50_ (μg/mL) ± SS)
**Control**	13.06 ± 1.94	-
**Negative control**	41.20 ± 5.12	-
**ELE**	38.19 ± 3.74	287.08 ± 8.51
**ELW**	35.48 ± 2.95	250.14 ± 6.95
**EFE**	27.01 ± 1.87	230.62 ± 8.04
**EFW**	31.74 ± 1.76	306.17 ± 8.13
**EBE**	44.51 ± 2.87	298.13 ± 8.07
**EBW**	19.32 ± 1.31**	149.56 ± 8.11
**CLE**	18.23 ± 1.34**	56.22 ± 4.94
**CLW**	21.12 ± 2.08**	68.95 ± 4.13
**CFE**	23.06 ± 1.08**	72.84 ± 6.47
**CFW**	29.14 ± 0.83**	92.08 ± 8.15
**CFrE**	31.26 ± 0.54*	111.49 ± 7.82
**CFrW**	37.09 ± 0.92*	135.48 ± 8.09
**Reference**	8.21 ± 0.46***	20.53 ± 0.98

S.E.M.: standard error meaning; *: *p* < 0.05; **p < 0.01; ***: *p* < 0.001.

ELE, 50% ethanolic extract of elderberry leaves; ELW, elderberry leaf water extract; EFE, 50% ethanolic extract of elderflowers; EFW, elderflower water extract; EBE, 50% ethanolic extract of elderberries; EBW, elderberry water extract; CLE, 70% ethanolic extract of chasteberry leaves; CLW, chasteberry leaf water extract; CFE, 70% ethanolic extract of chasteberry flower; CFW, chasteberry flower water extract; CFrE, 70% ethanolic extract of chasteberry fruit; CFrW, chasteberry fruit water extract. Bold values are statistically significant.

## 4 Discussion

Within the scope of our study, it was observed that CLE, CLW and CFE coded *V.agnus-castus* extracts had higher anti-obesity activities compared to other *V.agnus-castus* and *S.nigra* extracts. When the LC-HRMS analysis results were examined, it was observed that these CLE, CLW and CFE extracts, which have high obesity activity, contain kaempferol and luteolin-7-glucoside compounds at relatively high levels. The amount of luteolin-7-glycoside was determined as 1,720.26 mg/100 g, 690.49 mg/100 g and 653.04 mg/100 g while the amount of kaempferol was determined as 95.85 mg/100 g, 194.41 mg/100 g and 62.63 mg/100 g in CLE, CLW and CFE extracts, respectively. Luteolin and its most common derivative in plants luteolin-7-glucoside demonstrated anti-obesity properties, decreasing body and epididymal fat weight, as well as metabolic obesity-related complications, such as vascular dysfunction, in an *in vivo* investigation on high-fat diet (HFD) mice (Caporali et al., 2022). In a study conducted by Torres-Villarreal et al. (2019), it was reported that kaempferol inhibits the production of the Cebpa gene, which in turn reduces the amount of fat that accumulates in mature adipocytes by raising the levels of Pnpla2 and Lipe mRNA. This process occurs in 3T3-L1 cells during adipogenic differentiation. Hence, they concluded that kaempferol might have an anti-obesity effect. Bian et al. (2022) found that kaempferol protects against HFD-induced obesity through a combination of its prebiotic and anti-inflammatory properties. The anti-obesity effect observed in the studies we conducted is most likely caused by luteolin-7-glucoside and kaempferol, which are present in relatively high amounts in CLE, CLW and CFE extracts and have anti-obesity effects of their own.

The results obtained in the present study were consistent with the view that a high-fat diet leads to lipid accumulation in visceral tissues and triggers an increase in body weight and leptin levels (Chilliard et al., 2001; Milagro et al., 2006; Yang et al., 2006). Energy homeostasis is maintained by complex and dynamic procedures that interact with each other. Among these, leptin is an adipocyte-derived hormone that acts as a master regulator of food intake and energy homeostasis and exerts metabolic effects through specific receptors in the central nervous system and peripheral tissues (Houseknecht et al., 1998). This hormone also stimulates signals from adipose tissue for energy expenditure (Trayhurn et al., 1999). Residual leptin in plasma crosses the blood-brain barrier and, via hypothalamic receptors, inhibits the release of neuropeptide Y (NPY) in the nucleus accumbens, the body’s appetite control center. Thus, it causes a decrease in appetite, sympathetic nervous system activation and an increase in metabolic rate and energy expenditure (Schwartz and Seeley, 1997).

In a study by Yang et al. (2006), it was reported that there was a significant increase in serum LDL-C, TG and cholesterol levels in mice fed a high-fat diet for 12 weeks. The results of the study were in parallel with our study.

The thyroid gland mainly synthesizes T_4_, while the metabolically active T_3_ is mainly produced in extra-thyroidal tissues by enzymatic 5′-deiodination of T_4_ (Kelly, 2000). Thyroid hormones in the blood are mainly bound to plasma proteins, namely, albumin, thyroxine-binding globulin (TBG) and thyroxine-binding pre-albumin (TBPA), in equilibrium with free forms that can cross cellular membranes and are considered the active forms of thyroid hormones (Kaneko, 1997). Thyroid hormones (TH) stimulate thermogenesis by increasing ATP consumption in TH-dependent processes and decreasing the efficiency of ATP synthesis (Ortega et al., 2007). Thyroid hormones prevent proton binding by stimulating the uncoupling protein (UCP) in the inner membrane of mitochondria. Thus, it causes more energy consumption by providing heat release instead of ATP synthesis (Aslan et al., 2004). Studies have shown that high-fat diet is associated with increased T_4_ deiodination and increased T_4_ uptake by target cells (Benvenga and Robbins, 1998) and that fatty acids may differentially regulate pituitary T_3_ and T_4_ uptake (Everts et al., 1995). Therefore, the higher T_4_ uptake resulting from a high fat diet may result in normal serum T_4_ concentration, high serum T_3_ levels and high thyroid hormone action (Brito et al., 2006). Our study results showed that the test samples had no effect on T_3_ and T_4_ levels.

An increase in plasma insulin level leads to induction of lipogenesis in liver and adipose tissues and an increase in plasma leptin and ob gene mRNA levels (Maffei et al., 1995; Noriega-Lopez et al., 2007). Insulin hormone increases glucose entry into cardiac muscle, skeletal muscle and adipose tissues and the sensitivity of these tissues to insulin (Noyan, 1993). However, it causes the uptake of other nutrients such as free fatty acids and amino acids into the cell. Glucose is therefore oxidized during glycolysis, raising the ATP/ADP ratio in the cytoplasm. The high ATP/ADP ratio triggers the exocytosis of insulin-containing granules. Fatty acids also increase insulin secretion by increasing β-oxidation of fatty acids leading to a high ATP/ADP ratio or by activating some protein kinases. Hyperinsulinemia seen in long-term high-fat diet consumption is known to occur because of pancreatic hyperplasia, fatty acid intake and increased pancreatic β-cell glucose metabolism (Noriega-Lopez et al., 2007).

In conclusion, the *in vivo* effect of the test samples on plasma levels of some hormones (leptin, insulin, T_3_ and T_4_), lipids (triglycerides, HDL and LDL) and lipase enzyme was investigated. The results showed that test samples CLE, CLW and CFE caused a decrease in body weight of rats with obesity induced by feeding a high-fat diet. When compared with the negative control group, test samples CLE, CLW and CFE caused a decrease in serum LDL, triglyceride and leptin levels and an increase in HDL and adiponectin levels. However, it was determined that test samples coded CLE, CLW and CFE caused a decrease in serum lipase, TNF-α and IL-1ß levels. In line with the results obtained, it is thought that *V. agnus-castus* leaves and flowers may have beneficial effects in the treatment of obesity due to their luteolin-7-glucoside and kaempferol content. In light of the present study’s findings, the clinical application of *V. agnus-castus* leaves and flowers against obesity should be evaluated through controlled clinical trials. Furthermore, in future studies, the *in vivo* anti-obesogenic effects of luteolin-7-glycoside and kaempferol should first be investigated separately, and then the *in vivo* effects of these substances in combination should be investigated and it should be determined whether they produce a synergistic anti-obesogenic effect.

## Data Availability

The original contributions presented in the study are included in the article/Supplementary Material, further inquiries can be directed to the corresponding author.
