# Supplementary material for: Evaluation of the anti-obesity effect of Sambucus nigra L. (elderberry) and Vitex agnus-castus L. (chasteberry) extracts in high-fat diet-induced obese rats
Source: Front Pharmacol. 2024 Jul 11;15:1410854. doi: 10.3389/fphar.2024.1410854 (PMC11269222; doi:10.3389/fphar.2024.1410854)

Supplementary Material

# Supplementary Figures

**Supplementary Figure 1.** LC-HRMS chromatograms of ELE extract.


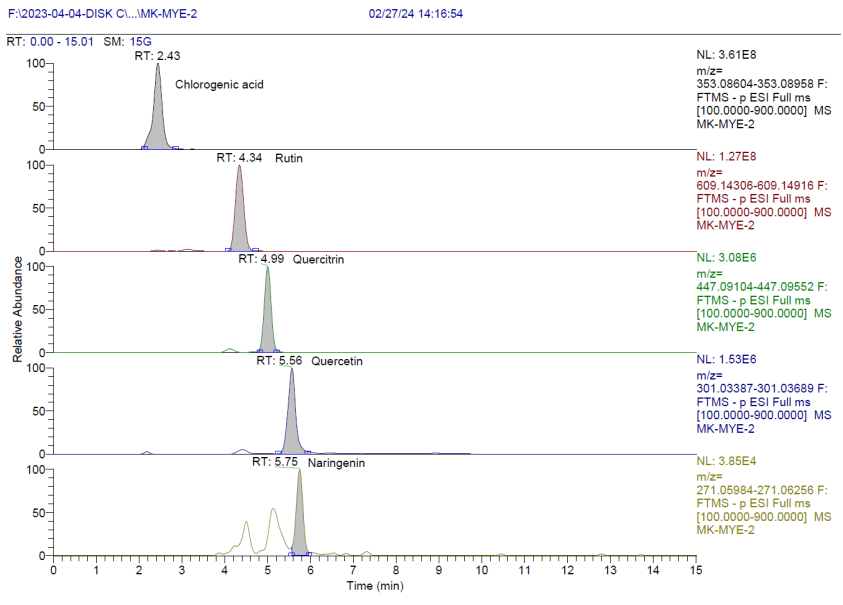


**Supplementary Figure 1.** LC-HRMS chromatograms of ELE extract (*continued*).


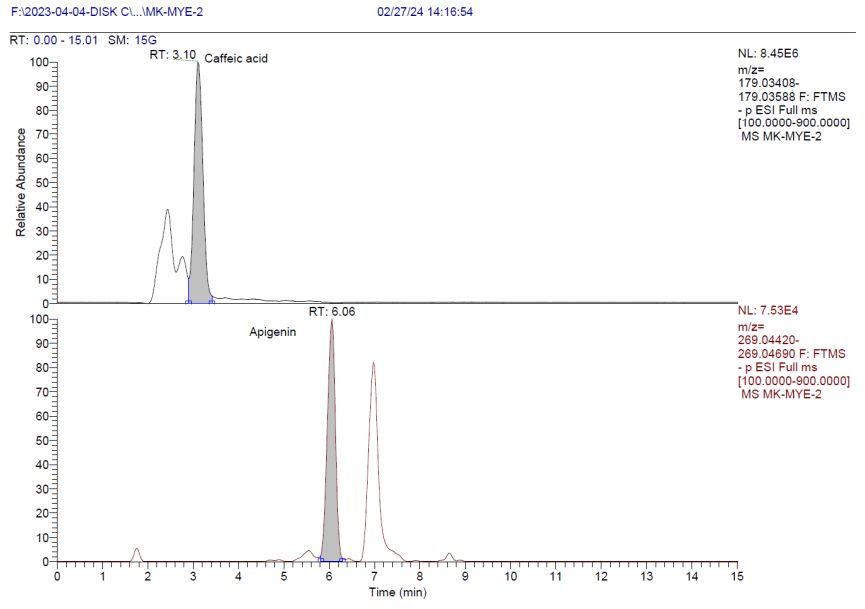


**Supplementary Figure 1.** LC-HRMS chromatograms of ELE extract (*continued*).


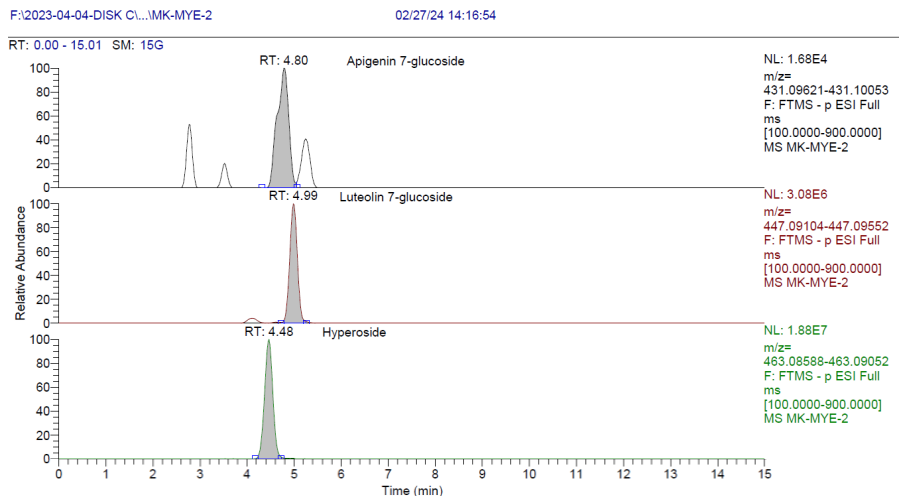


**Supplementary Figure 2.** LC-HRMS chromatograms of ELW extract.


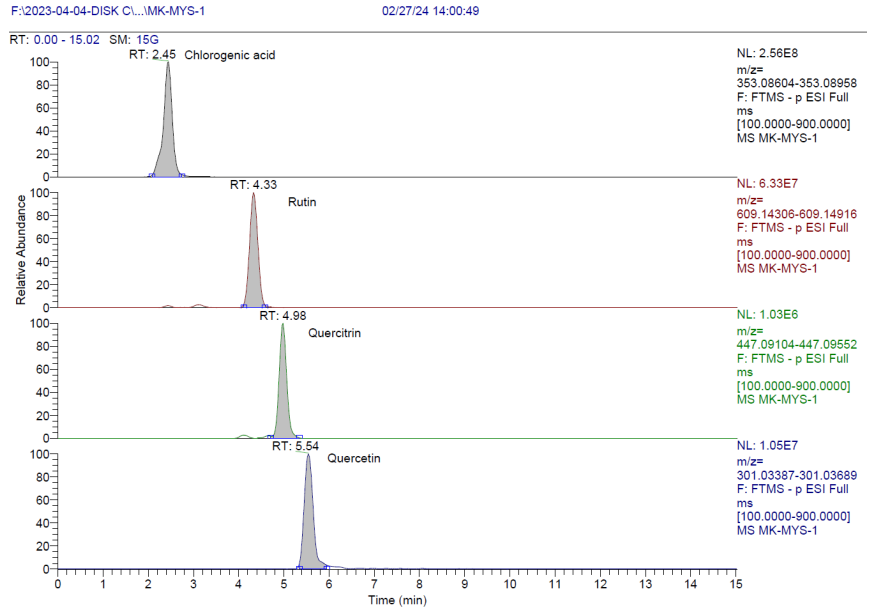


**Supplementary Figure 2.** LC-HRMS chromatograms of ELW extract (*continued*).


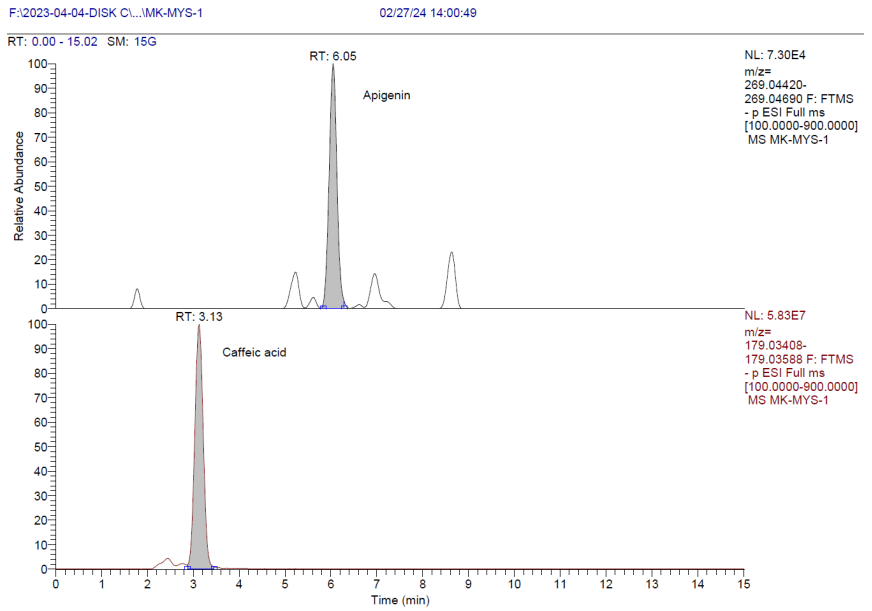


**Supplementary Figure 2.** LC-HRMS chromatograms of ELW extract (*continued*).


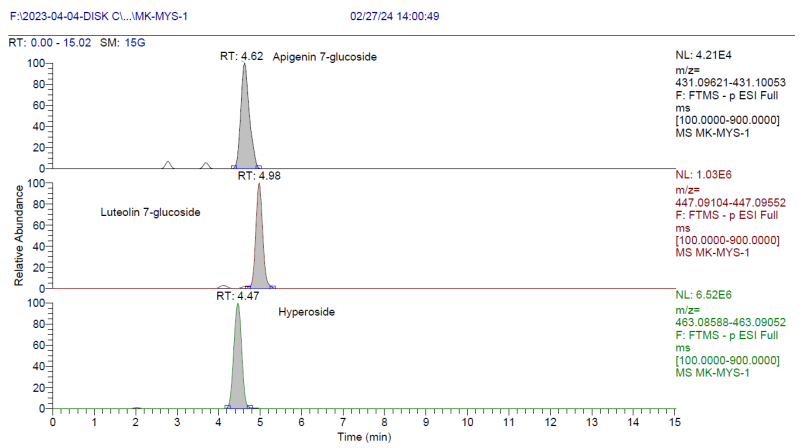


**Supplementary Figure 3.** LC-HRMS chromatograms of EFE extract.


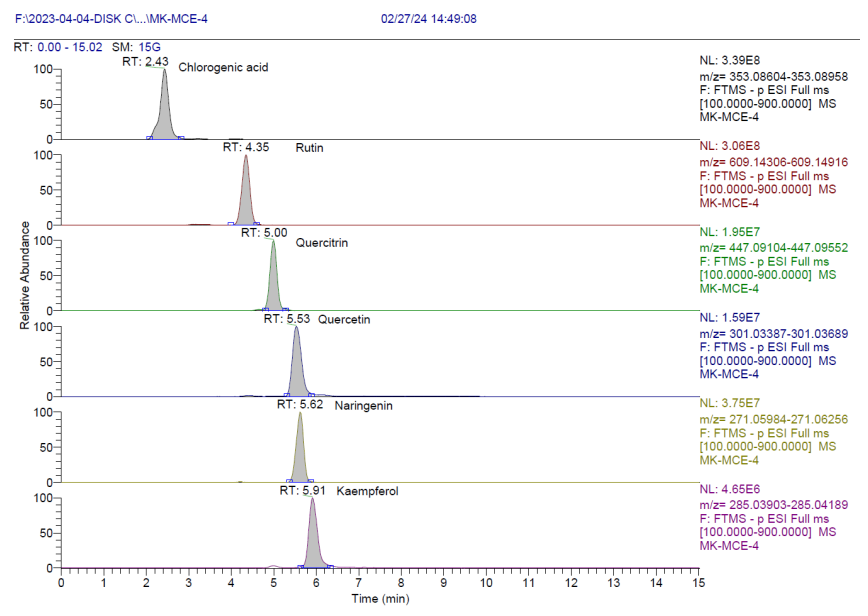


**Supplementary Figure 3.** LC-HRMS chromatograms of EFE extract (*continued*).


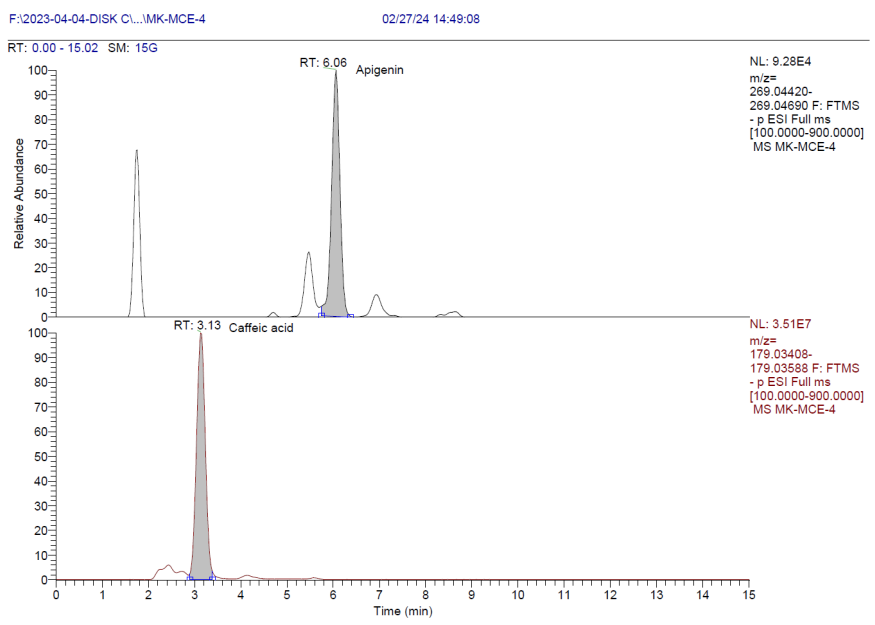


**Supplementary Figure 3.** LC-HRMS chromatograms of EFE extract (*continued*).


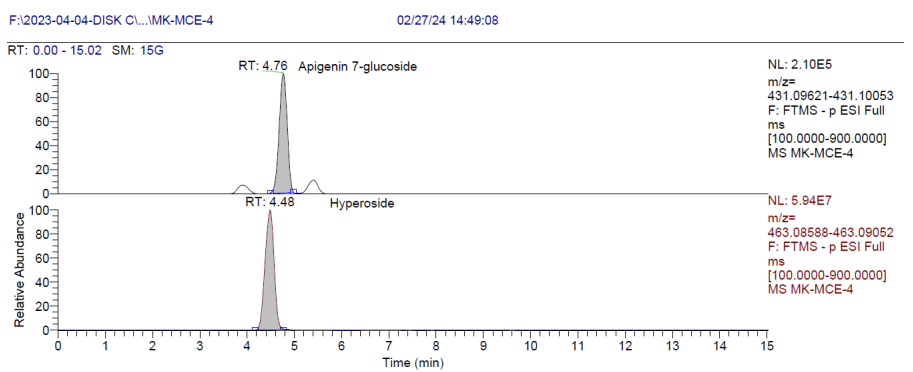


**Supplementary Figure 4.** LC-HRMS chromatograms of EFW extract.


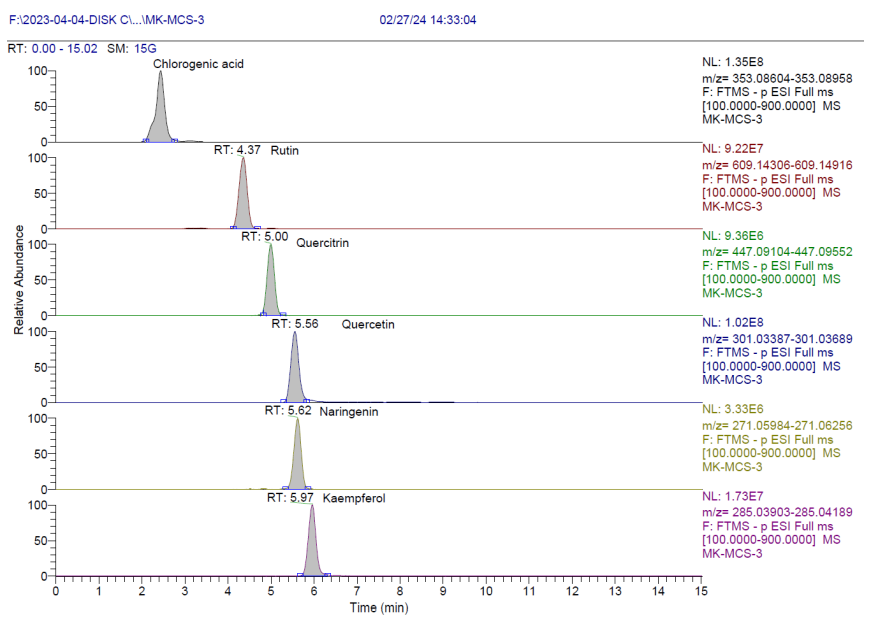


**Supplementary Figure 4.** LC-HRMS chromatograms of EFW extract (*continued*).


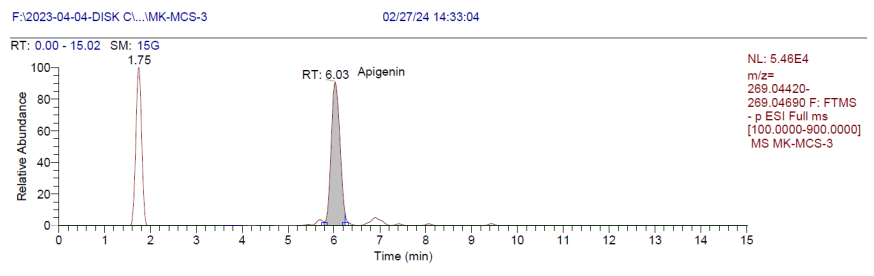


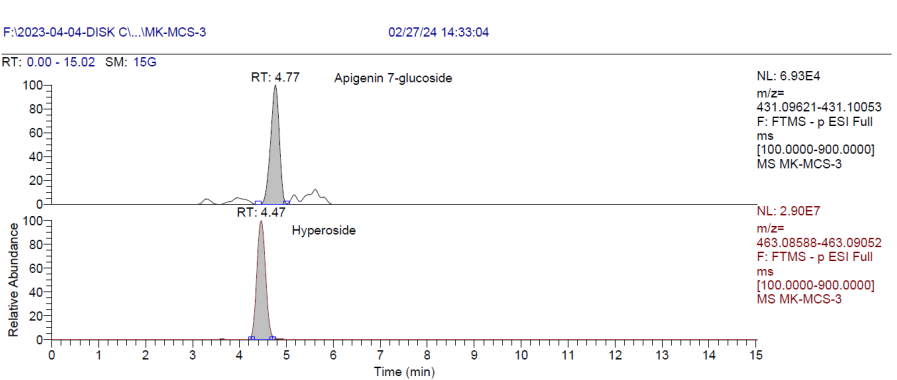


**Supplementary Figure 5.** LC-HRMS chromatograms of EBE extract.


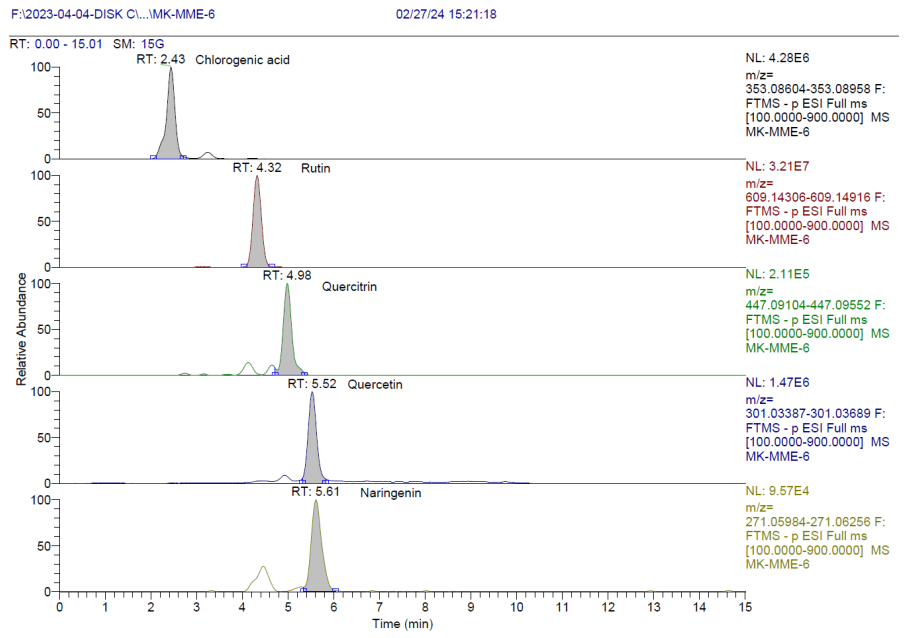


**Supplementary Figure 5.** LC-HRMS chromatograms of EBE extract (*continued*).


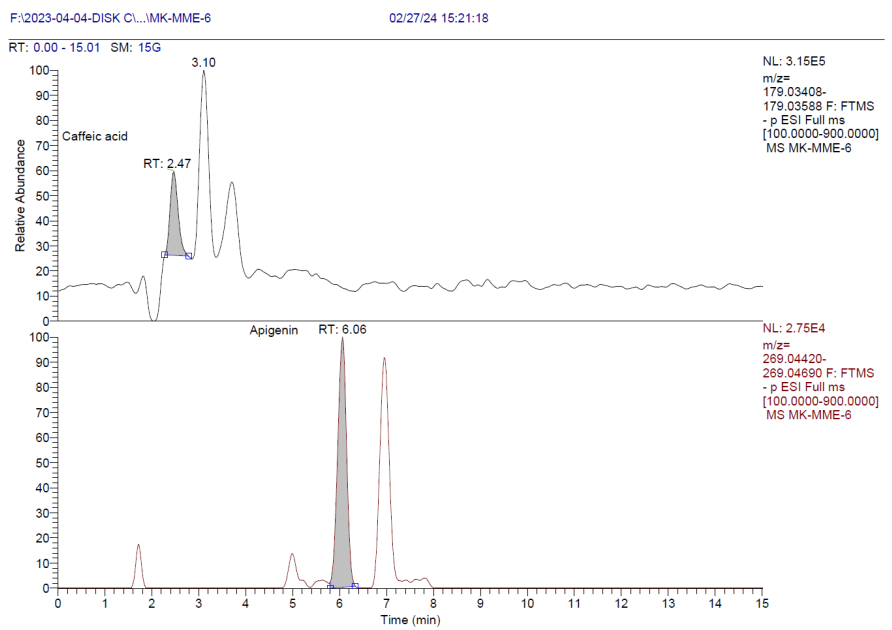


**Supplementary Figure 5.** LC-HRMS chromatograms of EBE extract (*continued*).


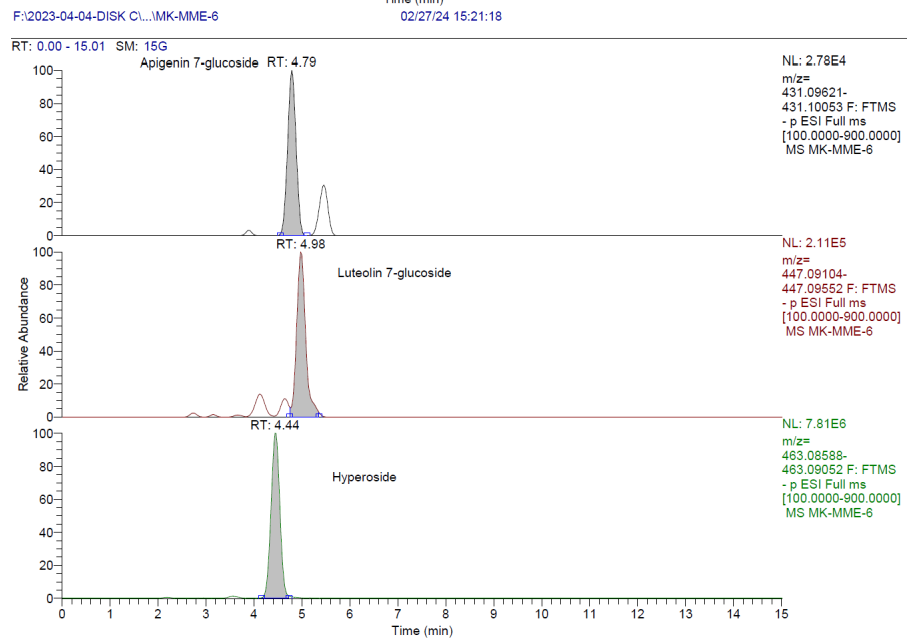


**Supplementary Figure 6.** LC-HRMS chromatograms of EBW extract.


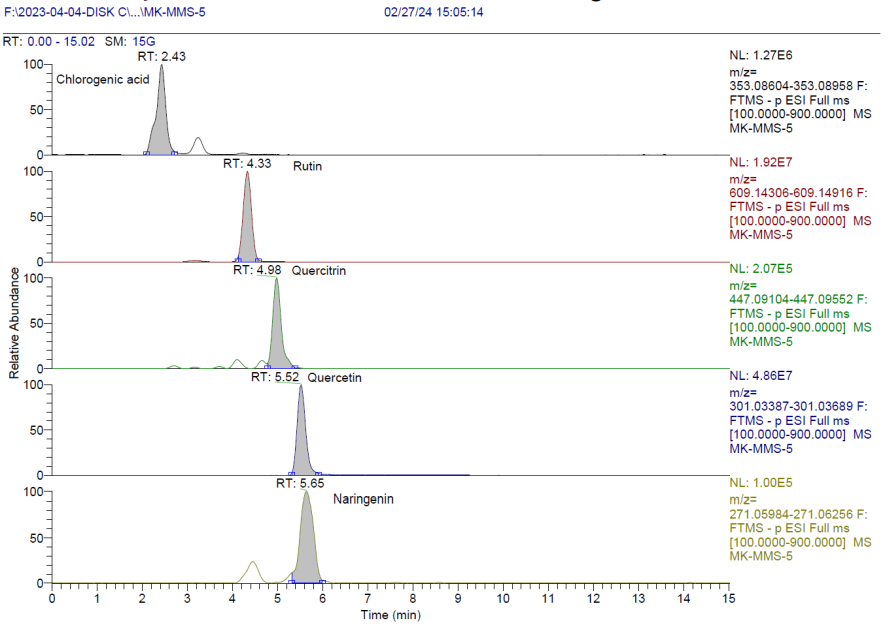


**Supplementary Figure 6.** LC-HRMS chromatograms of EBW extract (*continued*)


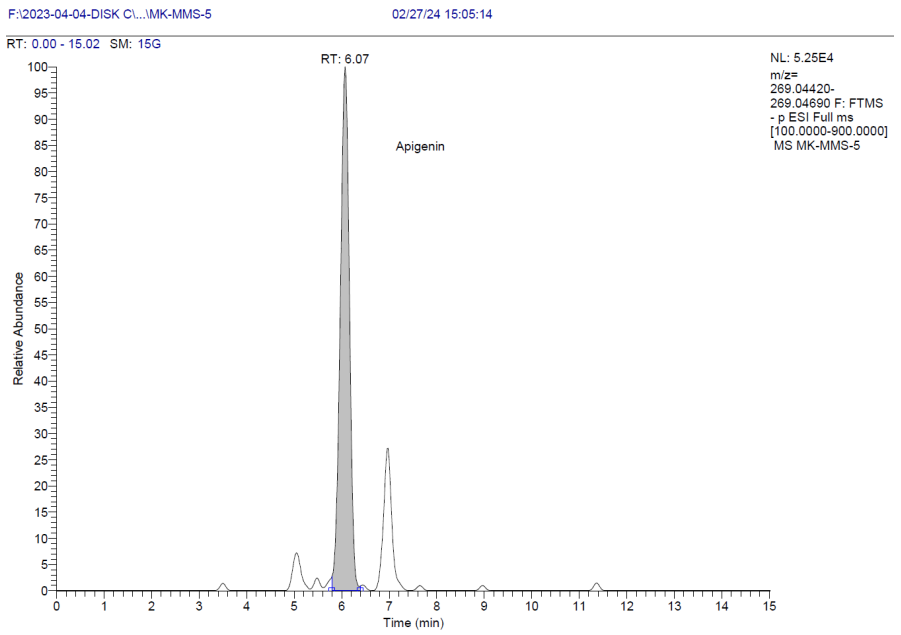


**Supplementary Figure 6.** LC-HRMS chromatograms of EBW extract (*continued*)


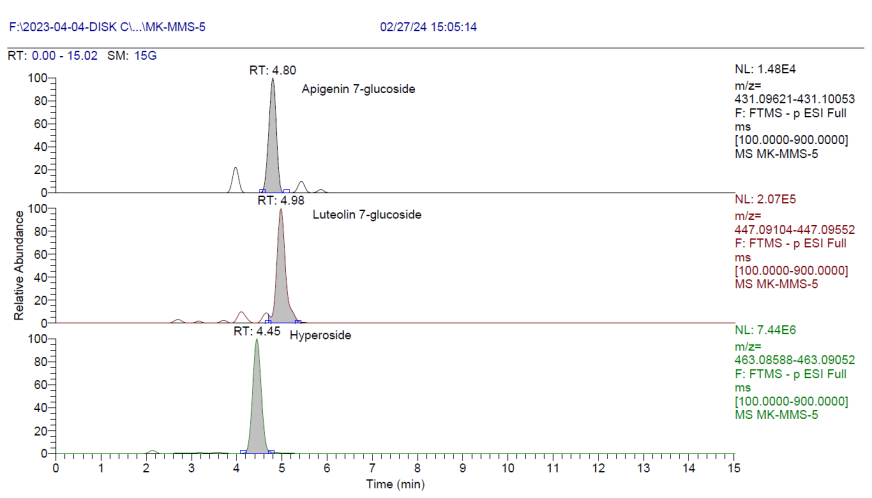


**Supplementary Figure 7.** LC-HRMS chromatograms of CLE extract


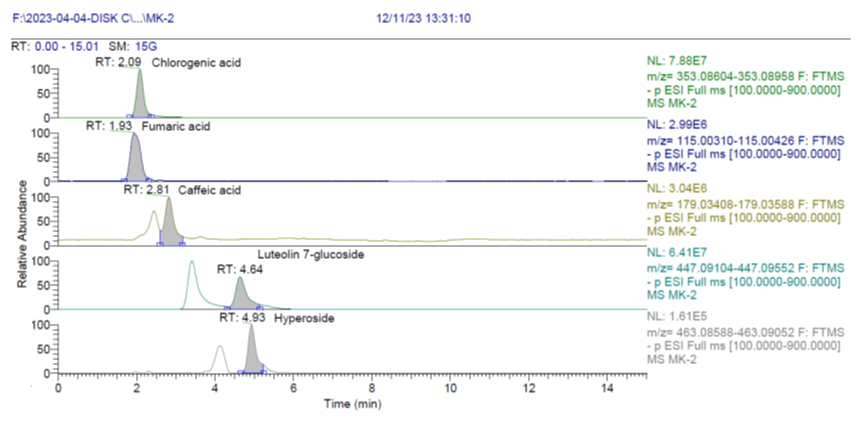


**Supplementary Figure 7.** LC-HRMS chromatograms of CLE extract (*continued*)


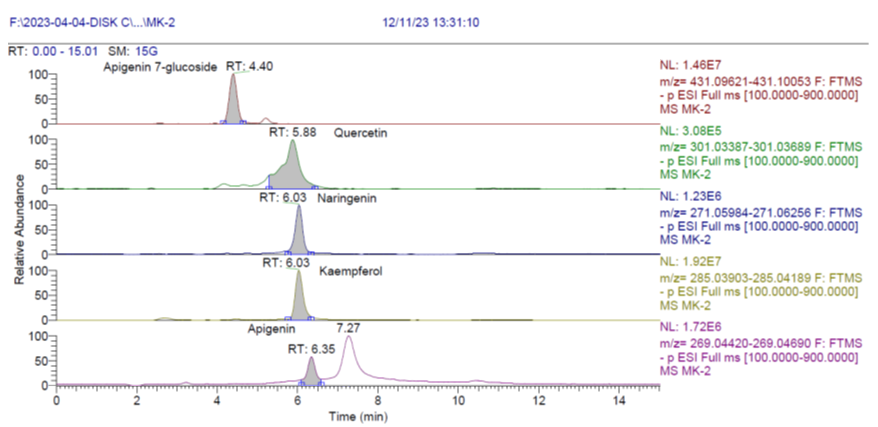


**Supplementary Figure 7.** LC-HRMS chromatograms of CLE extract (*continued*)


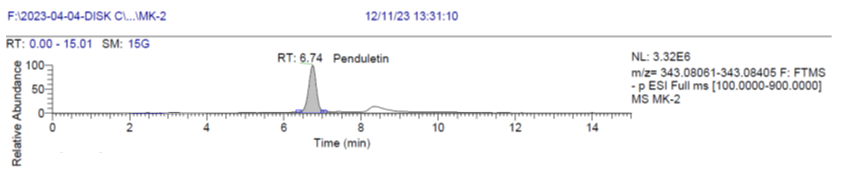


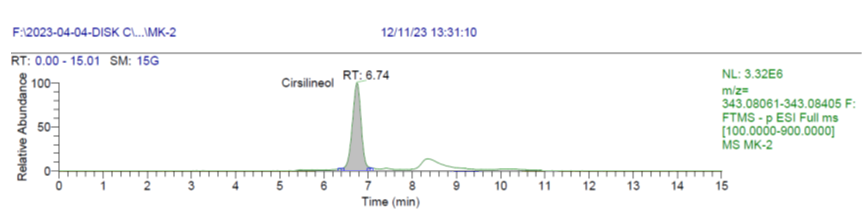


**Supplementary Figure 7.** LC-HRMS chromatograms of CLE extract (*continued*)


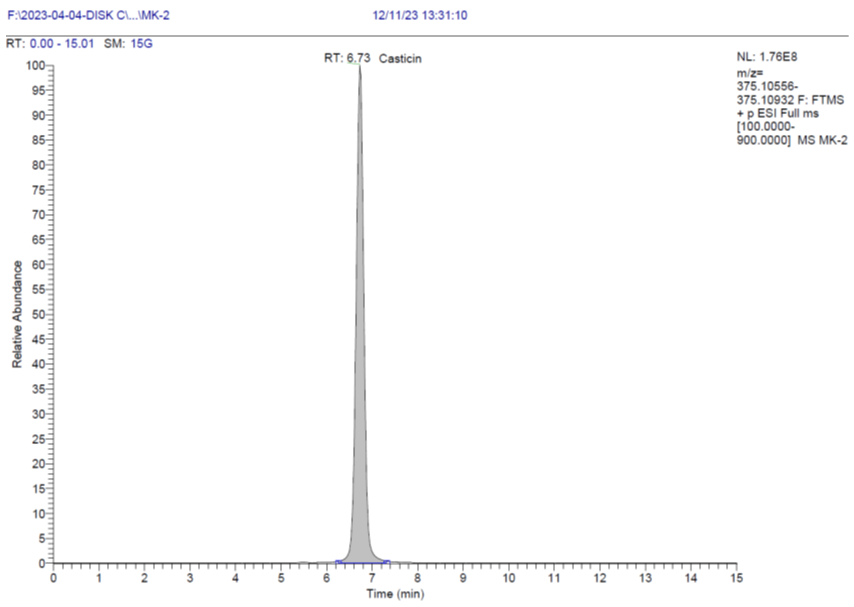


**Supplementary Figure 8.** LC-HRMS chromatograms of CLW extract


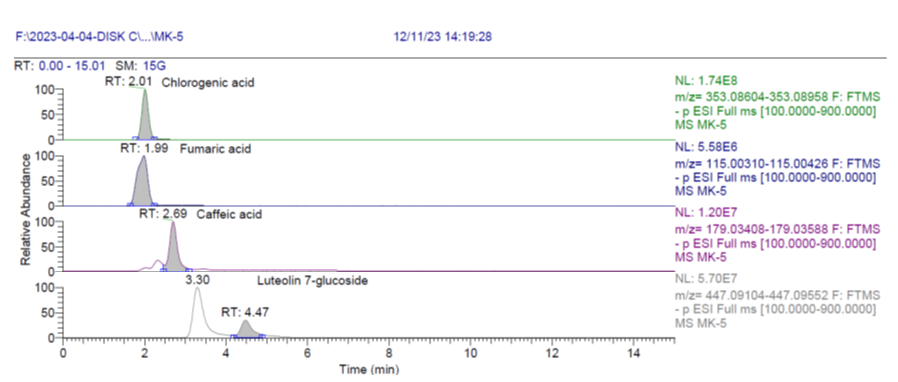


**Supplementary Figure 8.** LC-HRMS chromatograms of CLW extract (*continued*)


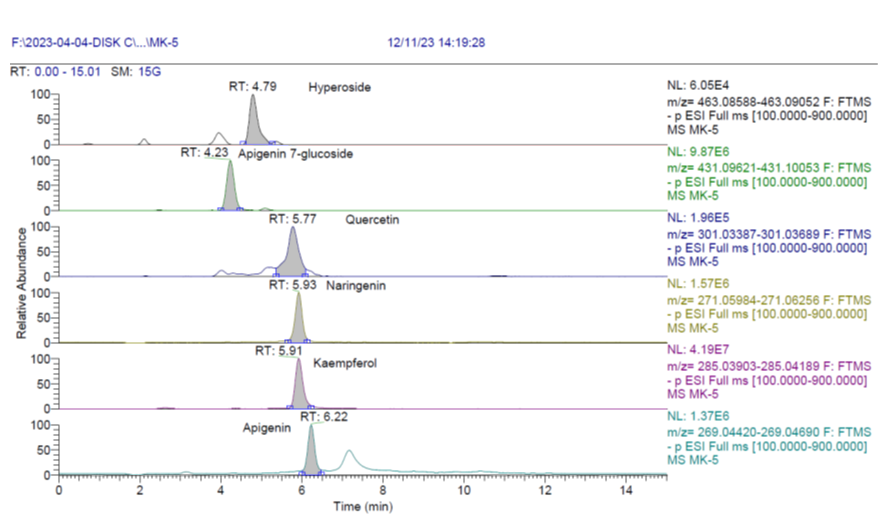


**Supplementary Figure 8.** LC-HRMS chromatograms of CLW extract (*continued*)


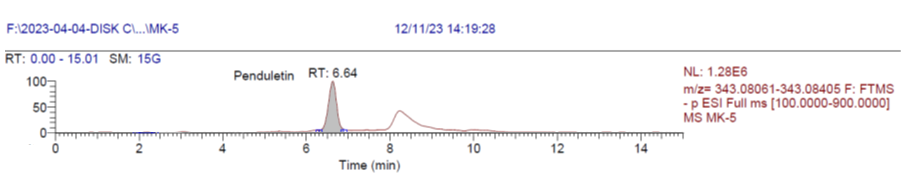


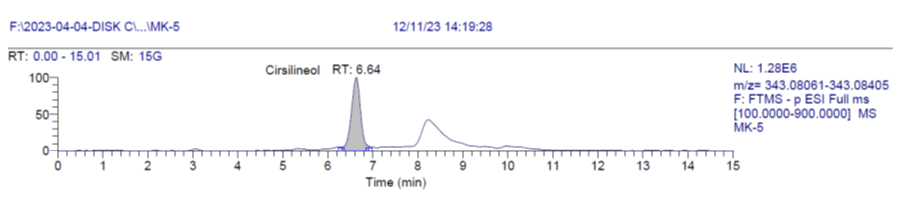


**Supplementary Figure 8.** LC-HRMS chromatograms of CLW extract (*continued*)


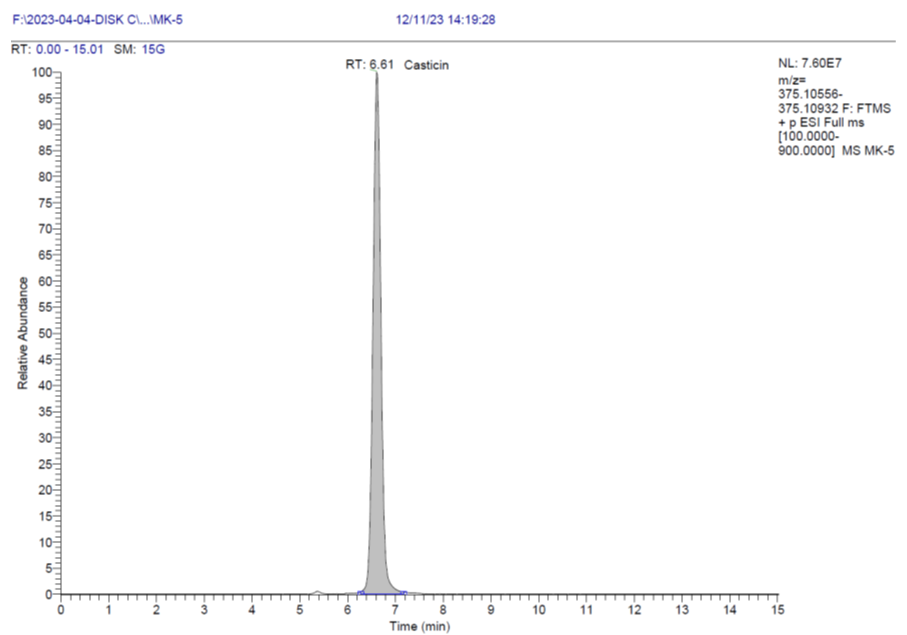


**Supplementary Figure 9.** LC-HRMS chromatograms of CFE extract


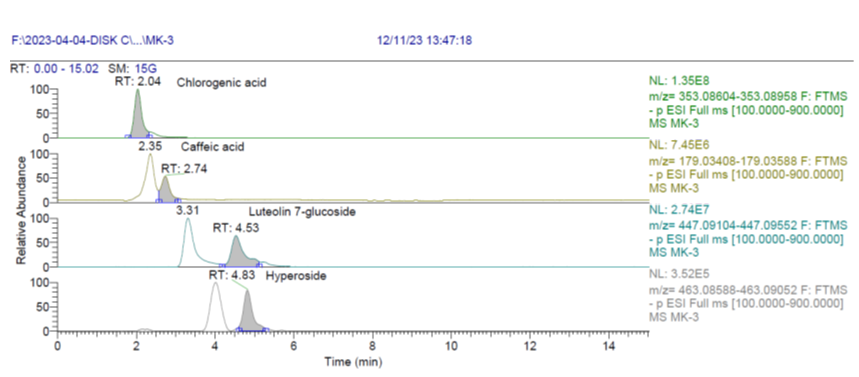


**Supplementary Figure 9.** LC-HRMS chromatograms of CFE extract (*continued*)


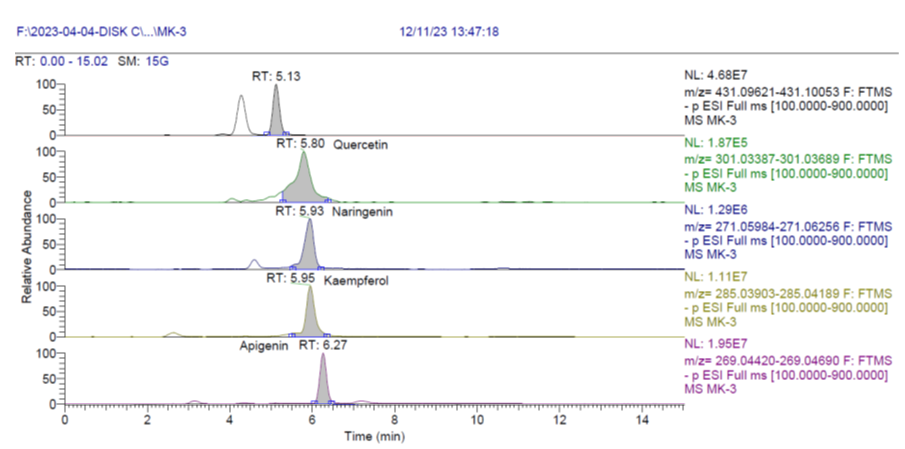


**Supplementary Figure 9.** LC-HRMS chromatograms of CFE extract (*continued*)


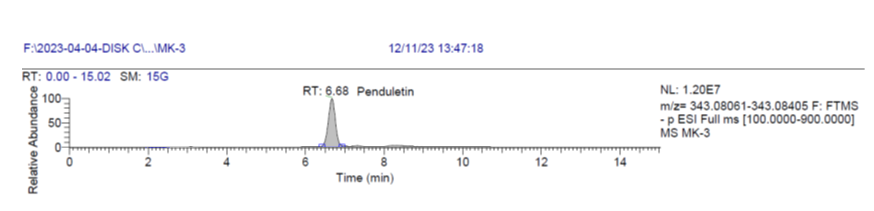


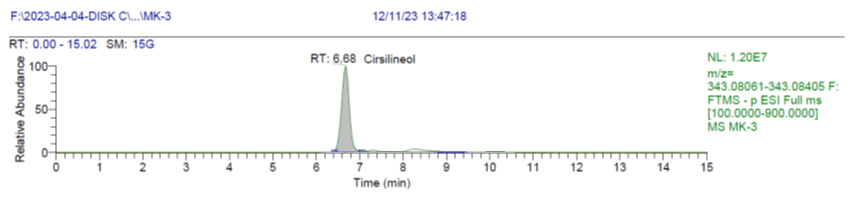


**Supplementary Figure 9.** LC-HRMS chromatograms of CFE extract (*continued*)


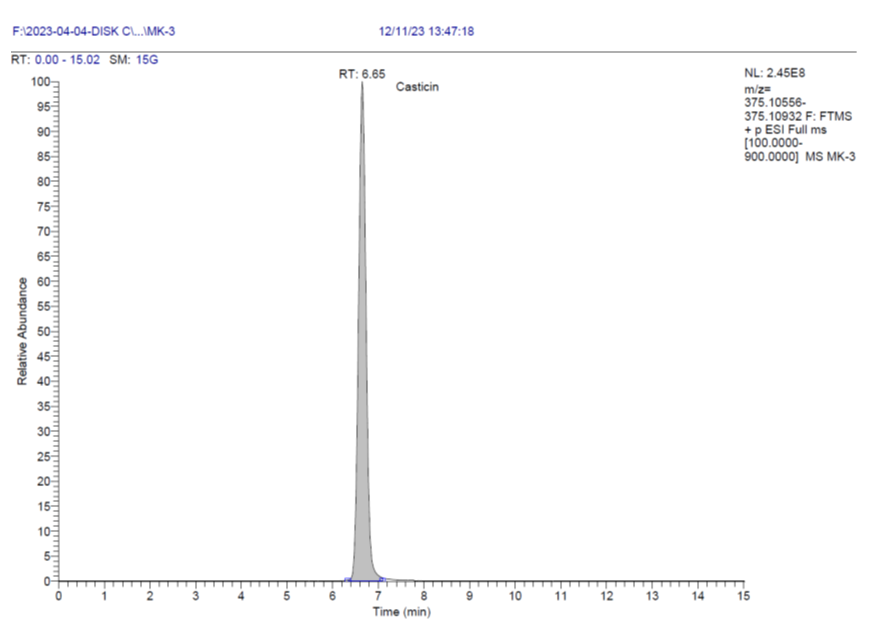


**Supplementary Figure 10.** LC-HRMS chromatograms of CFW extract


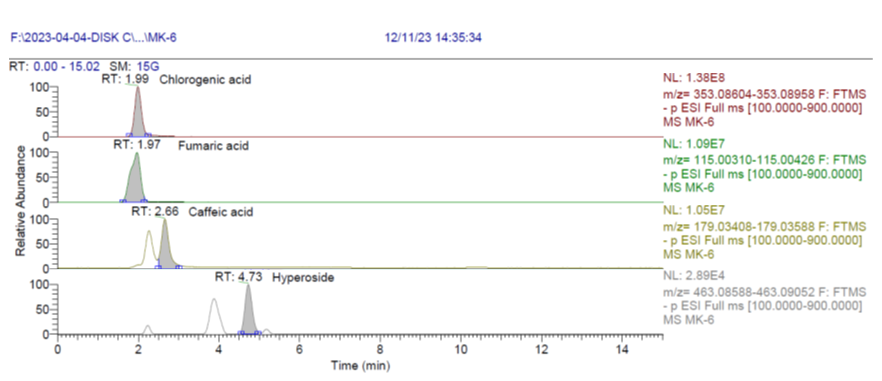


**Supplementary Figure 10.** LC-HRMS chromatograms of CFW extract (*continued*)


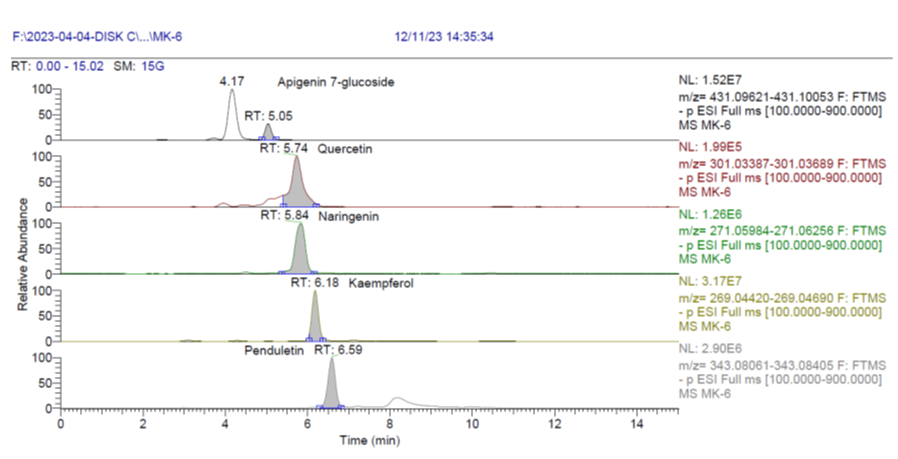


**Supplementary Figure 10.** LC-HRMS chromatograms of CFW extract (*continued*)


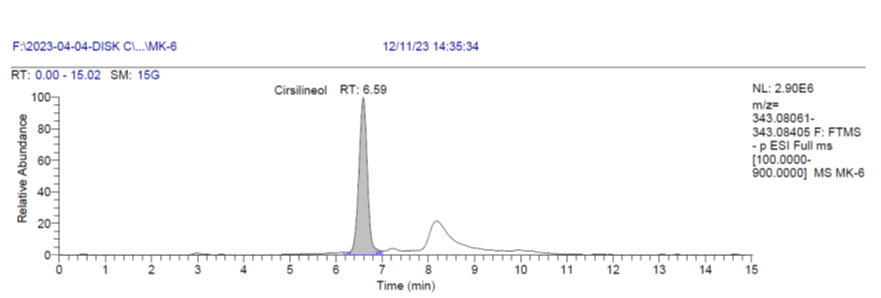


**Supplementary Figure 10.** LC-HRMS chromatograms of CFW extract (*continued*)


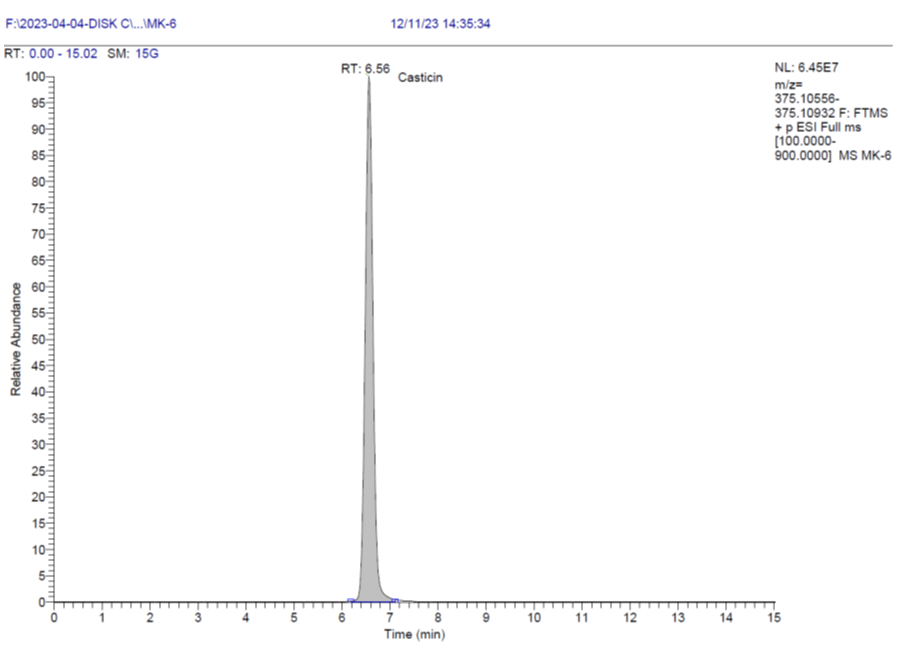


**Supplementary Figure 11.** LC-HRMS chromatograms of CFrE extract


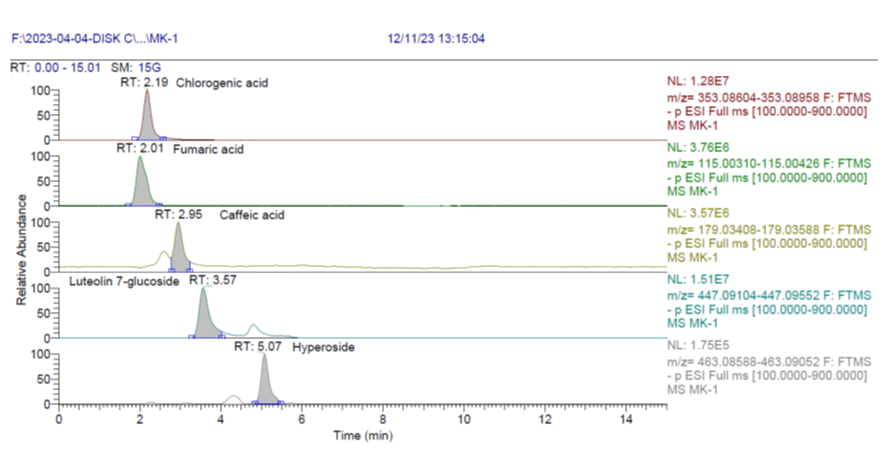


**Supplementary Figure 11.** LC-HRMS chromatograms of CFrE extract (*continued*)


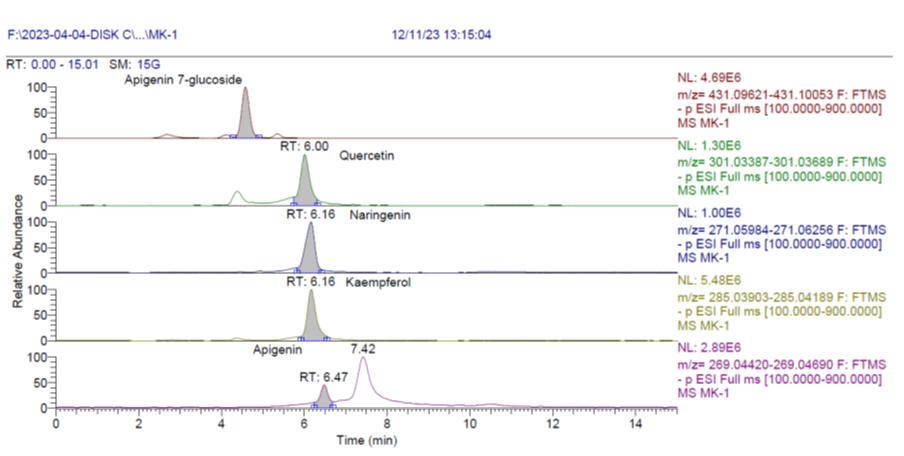


**Supplementary Figure 11.** LC-HRMS chromatograms of CFrE extract (*continued*)


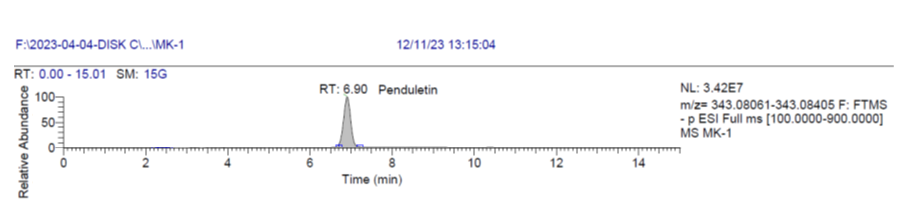


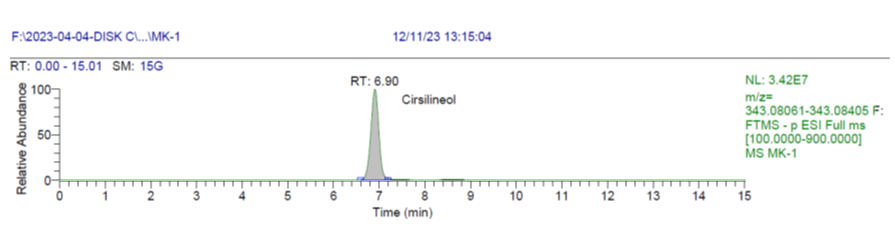


**Supplementary Figure 11.** LC-HRMS chromatograms of CFrE extract (*continued*)


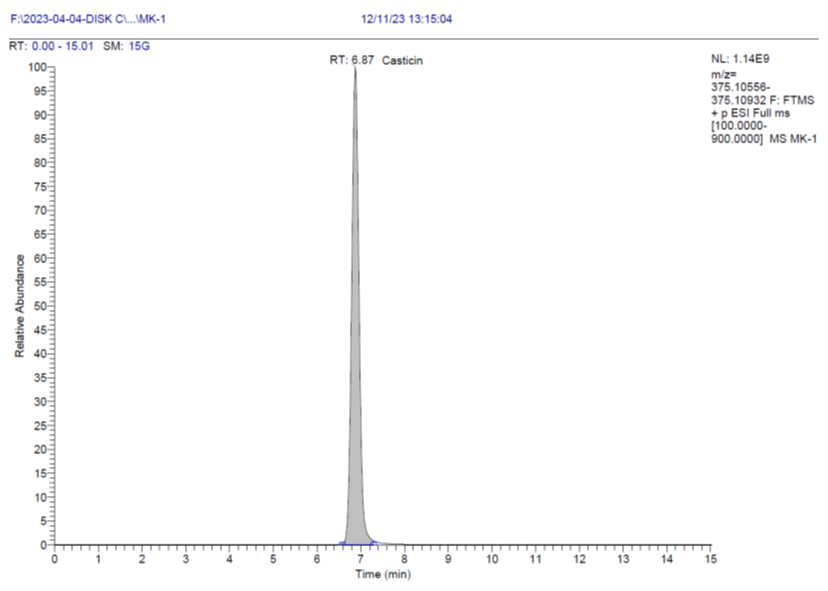


**Supplementary Figure 12.** LC-HRMS chromatograms of CFrW extract


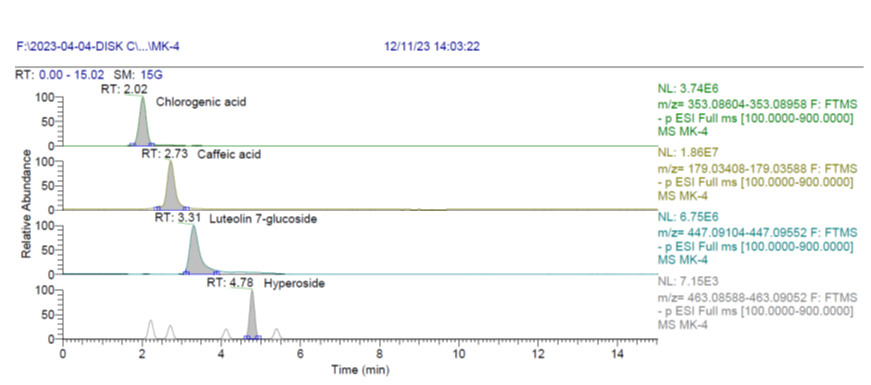


**Supplementary Figure 12.** LC-HRMS chromatograms of CFrW extract (*continued*)


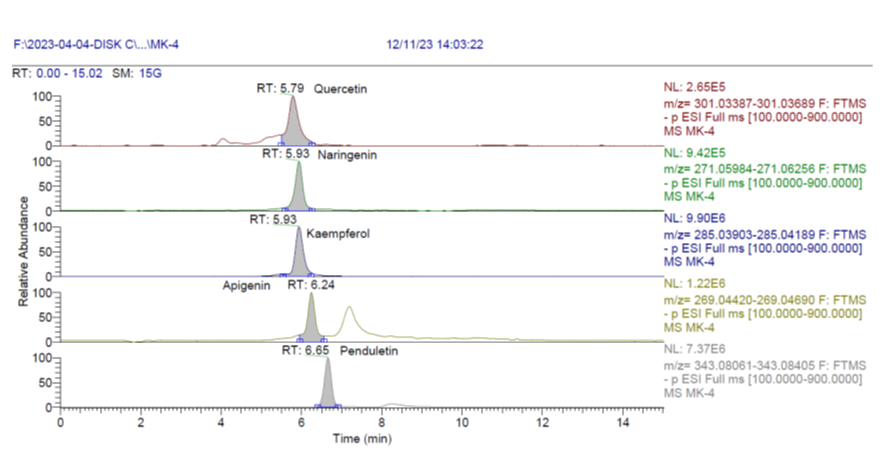


**Supplementary Figure 12.** LC-HRMS chromatograms of CFrW extract (*continued*)


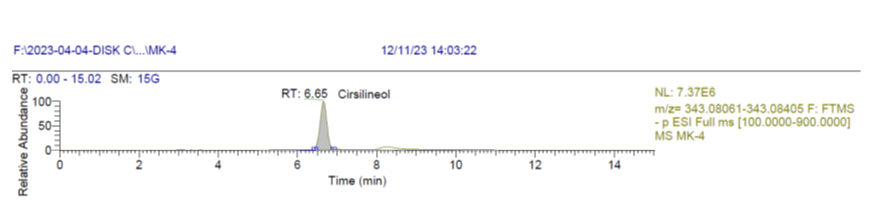


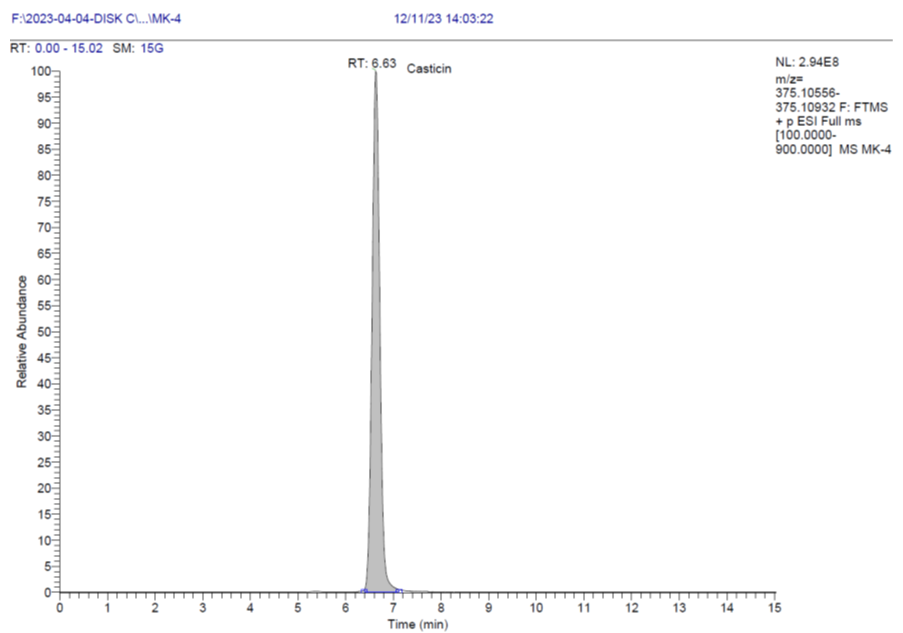

Supplement: Supplementary file 1 [file Table1.DOCX]
